# ﻿A catalog of Cassidinae leaf beetles (Coleoptera, Chrysomelidae) collection deposited in the Insect Collection of the College of Agriculture, Yangtze University, China

**DOI:** 10.3897/zookeys.1252.139438

**Published:** 2025-09-19

**Authors:** Chaokun Yang, Liang Zhang, Ping Wang, Guanglin Xie, Wenkai Wang

**Affiliations:** 1 Institute of Entomology, College of Agriculture, Yangtze University, Jingzhou, Hubei, 434025, China Yangtze University Jingzhou China; 2 MARA Key Laboratory of Sustainable Crop Production in the Middle Reaches of the Yangtze River (Co-Construction by Ministry and Province), College of Agriculture, Yangtze University, Jingzhou 434025, China Yangtze University Jingzhou China

**Keywords:** Hispine beetles, new record, specimen collections, tortoise beetles

## Abstract

A catalog is presented of species of the subfamily Cassidinae (Coleoptera, Chrysomelidae) deposited in the Insect Collection of the College of Agriculture, Yangtze University in Jingzhou, Hubei, China. A complete list of all specimens for each species is provided, together with the label data. A total of 1,207 adult specimens of Cassidinae were examined, representing 53 species, 13 genera, and six tribes. Fourteen species are recorded in Hubei Province for the first time: Aspidomorpha (s. str.) transparipennis (Motschulsky, 1860), *Basiprionota gressitti* Medvedev, 1957, *Callispa dimidiatipennis* Baly, 1858, *Cassida australica* (Boheman, 1855), *C. expansa* Gressitt, 1952, *C. juglans* Gressitt, 1942, *C. pallidicollis* Boheman, 1856, *C. postarcuata* (Chen & Zia, 1964), *C. quinaria* (Chen & Zia, 1964), *C. rati* Maulik, 1923, *C. sigillata* (Gorham, 1885), Glyphocassis (Hebdomecosta) spilota (Gorham, 1885), *Hispellinus chinensis* Gressitt, 1950, and *H. moerens* (Baly, 1874). Five species are newly recorded from Chongqing: Aspidomorpha (s. str.) difformis (Motschulsky, 1860), *Cassida sauteri* (Spaeth, 1913), *C. vespertina* Boheman, 1862, Gly. (Hebdomecosta) spilota (Gorham, 1885), and *Dactylispa chinensis* Weise, 1905. Furthermore, *Callispa almora* Maulik, 1923 is new to Zhejiang Province, *Platypria aliena* Chen & Sun, 1962 is new to Guangxi Zhuang Autonomous Region, and *Cassida rubiginosa* Müller, 1776 is new to Sichuan Province.

## ﻿Introduction

The subfamily Cassidinae Gyllenhal, 1813 sensu lato includes approximately 6,400 species (Borowiec and Świętojańska 2012–2024; Staines 2015; Sekerka 2020; Hobern et al. 2021), accounting for approximately 16% of the species diversity within the leaf beetles (Chrysomelidae Latreille, 1802) and representing the second richest clade, after Galerucinae Latreille, 1802 (Chaboo 2007). Historically, the Cassidinae (tortoise beetles) and Hispinae (hispine beetles) were treated as separate subfamilies (Seeno and Wilcox 1982). The current combination of tortoise beetles and hispine beetles is relatively recent (Staines 2002).

Approximately 500 species in 52 genera from 14 tribes have been recorded in China (Gressitt 1938, 1950, 1952; Gressitt and Kimoto 1963; Chen et al. 1986; Hua 2002; Qi et al. 2008; Lee et al. 2011, 2012, 2016; Borowiec and Świętojańska 2012–2024; Staines 2015; Sekerka et al. 2016; Liao et al. 2018b; Yang et al. 2023). In recent years, surveys of Cassidinae in various regions have revealed several newly recorded species in China, as well as new provincial or autonomous region records (Qi et al. 2008; Lee et al. 2012; Liao et al. 2018a; Liu et al. 2019; Yang et al. 2023, 2024). However, there are still a large number of specimens scattered and stored in multiple collections that have not been studied, and distribution records for many species are also lacking. In addition, the few monographs and specialized lists on Cassidinae in China have rarely been updated (Liu 1936; Gressitt 1938, 1939a, b, 1950, 1952; Gressitt and Kimoto 1963; Chen et al. 1986; Hua 2002; Lee et al. 2016).

The Insect Collection of the College of Agriculture, Yangtze University (YZU), located in Jingzhou City, Hubei Province, China, is one of the largest insect collections in Central China. Its predecessor, the Insect Specimen Room of Hubei Agricultural College, was established in the 1980s, with a collection focused on insect specimens from the western region of Hubei, encompassing areas such as Shennongjia National Park, Dalaoling National Nature Reserve, Houhe National Nature Reserve, and other national nature reserves. It currently houses over 650,000 insect specimens. The earliest Cassidinae specimens, *Cassida piperata* Hope, 1842, housed in the collection, date back to 1987. Since then, many Cassidinae specimens have been collected. As of September 2024, more than 1,200 adult specimens of Cassidinae have been accumulated in the collection, however, no information is available on their larval stages or host plants.

This paper provides a catalog of the Cassidinae beetles preserved in the YZU and updates the distribution data for some Cassidinae beetles in China.

## ﻿Material and methods

The Cassidinae material examined in this study was collected from nine provinces, autonomous regions, and municipalities and was deposited in the YZU. Adult Cassidinae specimens were identified using the available diagnostic keys (Chen et al. 1986), online resources (Staines 2012; Borowiec and Świętojańska 2012–2024), and photos of type species. Cassidinae classification, taxonomic names and distribution primarily follow Chen et al. (1986); Borowiec and Świętojańska (2012–2024); Staines (2015); Bezděk and Sekerka (2024). Taxa in the catalog below are arranged alphabetically, by tribe, genus, and species.

Photographs were captured using a Canon 5D Mark II DSLR camera with a Canon EF 100 mm f/2.8 L IS USM lens and Laowa 25 mm f/2.8 2.5–5X Ultra Macro lens. Multiple images were taken at different focal planes with the lowest aperture, and the images were subsequently stacked and composed using Helicon Focus software.

All label data were transcribed verbatim as appearing on the respective labels. The complete label information of each specimen is presented as follows: collection location, date of collection, altitude, latitude, longitude, collector, and specimen number. Not all Cassidinae specimens have complete information, and without such information, the position will be omitted from the label, with a semi-colon to separate distinct collecting events, i.e., Sichuan: FPV, 26 VII 2024, 1,717.2 m a.s.l., 32.686682°N, 104.076656°E, Chaokun Yang leg. [1 spec., CS-62]; Hubei; LPT, 15 VII 2008, Bo Zhan leg. [1 spec., A-265]; Guizhou: NPT: 18 VIII 2024, Chaokun Yang leg. [3 spec., CS-308–CS-310]. New distribution records are marked with ‘*’.

### ﻿Abbreviations of locality used in the text

#### ﻿Chongqing Municipality

**JFS** Jinfoshan National Nature Reserve, Chongqing

**MBV** Miaoba Village, Wangping Town, Chongqing

#### ﻿Guangdong Province

**NKS** Nankunshan National Forest Park, Longmen County, Guangdong

#### ﻿Guangxi Zhuang Autonomous Region (Guangxi)

**BAT** Baoan Township, Du’an County, Guangxi

**BDV** Baidan Village, Chuanshan Town, Huanjiang, Guangxi

**BNV** Baini Village, Xinhua Town, Leye County, Guangxi

**CFV** Chaofang Village, Baping Township, Nandan County, Guangxi

**DPV** Dianping Village, Leye County, Guangxi

**JG** Jiugong, Bagong Town, Jinchengjiang District, Guangxi

**LLV** Liangli Village, Sanmenhai Town, Fengshan County, Guangxi

**LWV** Leweng Village, Xinhua Town, Leye County, Guangxi

**PTV** Pantu Village, Yalong Township, Dahua County, Guangxi

**SHV** Sihe Village, Gantian Town, Leye County, Baise City, Guangxi

**SLT** Shangletun, Yuhuan Village, Huanjiang County, Guangxi

**XCV** Xinchang Village, Yachang Township, Leye County, Guangxi

**XLV** Xinglong Village, Fengcheng Town, Fengshan County, Guangxi

**YG** Yigou, Yachang Township, Baise City, Guangxi

#### ﻿Guizhou Province

**CMC** Cangmuchi, Huangtu Town, Yanhe County, Guizhou

**HTT** Huangtu Town, Yanhe County, Guizhou

**HTW** Hetaowan, Yinjiang County, Guizhou

**KKS** Kuankuoshui National Nature Reserve, Suiyang County, Guizhou

**NPT** Niupitang, Huangtu Town, Yanhe County, Guizhou

**TLV** Tuanlong Village, Ziwei Town, Yinjiang County, Guizhou

**XJT** Xinjing Town, Yanhe County, Guizhou

**YK** Yankou, Suiyang County, Guizhou

#### ﻿Hainan Province

**DLS** Diaoluoshan National Forest Park, Hainan

**JFL** Jianfengling National Nature Reserve, Ledong County, Hainan

#### ﻿Hubei Province

**BDC** Badong County, Hubei

**BJMV** Bajiaomiao Village, Shennongjia, Hubei

**BYT** Bingying Town, Zhuxi County, Hubei

**CLPT** Changleping Town, Wufeng County, Hubei

**DLLFF** Dalaoling Forest Farm, Yichang, Hubei

**DLLNNR** Dalaoling National Nature Reserve, Hubei

**FXT** Fengxi Town, Zhuxi County, Hubei

**HBAU** Hubei Agricultural University, Hubei

**HH** Houhe National Nature Reserve, Wufeng County, Hubei

**HHT** Hehua Town, Yuanan County, Hubei

**HLT** Huangliang Town, Xingshan County, Hubei

**HMCV** Hanmachi Village, Yuguan Town, Wufeng County, Hubei

**HPT** Hongping Town, Shennongjia, Hubei

**HWT** Huiwan Town, Zhuxi County, Hubei

**HZS** Huzhuashan Forest Farm, Jingshan City, Hubei

**JJYT** Jiangjiayan Town, Zhuxi County, Hubei

**JLC** Taihu Lake, Jiangling County, Hubei

**JPV** Jiangping Village, Xiaping Town, Hefeng County, Hubei

**JZ** Jingzhou City, Hubei

**LLT** Lvlin Town, Jingshan County, Hubei

**LMHV** Longmenhe Village, Xingshan County, Yichang City, Hubei

**LPT** Langping Town, Changyang County, Yichang City, Hubei

**LWCV** Longwangchong Village, Hejiaping Town, Yichang, Hubei

**MPT** Maoping Town, Yuan’an County, Hubei

**MYT** Muyu Town, Shennongjia, Hubei

**NJHT** Niejiahe Town, Yidu City, Hubei

**NMPV** Nanmuping Village, Changyang County, Hubei

**NTT** Niehe Town, Yidu County, Hubei

**NYT** Nanyang Town, Xingshan County, Hubei

**QGPV** Qinggangping Village, Hejiaping, Changyang County, Hubei

**QJPV** Qingjiangping Village, Wuduhe Town, Yichang City, Hubei

**QXT** Quanxi Town, Zhuxi County, Hubei

**RHPT** Renheping Town, Wufeng County, Hubei

**SBT** Songbai Town, Shennongjia, Hubei

**SDSV** Sandongshui Village, Changyang County, Hubei

**SPT** Shuiping Town, Zhuxi County, Hubei

**SQPV** Shiqiaoping Village, Yesanguan Town, Badong County, Hubei

**SS** Songshan Scenic Area, Yidu City, Hubei

**SYPV** Songyuanping Village, Changyang County, Hubei

**SYST** Shuiyueshi Town, Xingshan County, Hubei

**SZPT** Shengziping Township, Wufeng County, Hubei

**TBT** Tianbao Township, Zhuxi County, Hubei

**TYT** Taoyuan Township, Zhuxi County, Hubei

**TZS** Tianzhushan, Changyang County, Hubei

**WDHT** Wuduhe Town, Yichang City, Hubei

**WJV** Wangjia Village, Hehua Town, Yuan’an County, Hubei

**WSFF** Wenshui Forest Farm, Shennongjia, Hubei

**XBT** Xiangba Township, Zhuxi County, Hubei

**XBPT** Xiabaoping Township, Yichang City, Hubei

**XPV** Xiping Village, Qiaoshang Township, Fang County, Hubei

**XRX** Xianrenxi, Yichang City, Hubei

**XST** Xinshi Town, Jingshan City, Hubei

**XZT** Xinzhou Town, Zhuxi County, Hubei

**YGT** Yuguan Town, Wufeng County, Hubei

**YJFF** Yangji Forest Farm, Yangji Town, Jingshan City, Hubei

**YJG** Yujiagou, Badong County, Hubei

**YPT** Yangping Town, Yuanan County, Hubei

**YSGT** Yesanguan Town, Badong, Hubei

**ZFT** Zhongfeng Town, Zhuxi County, Hubei

**ZGW** Zhongguwan, Changyang County, Hubei

#### ﻿Sichuan Province

**FPV** Fengping Village, Songpan County, Sichuan

**SG** Sigou, Shuanghe Village, Songpan County, Sichuan

#### ﻿Yunnan Province

**DL** Dali City, Yunnan

#### ﻿Zhejiang Province

**FYS** Fengyangshan National Nature Reserve, Longquan City, Zhejiang

## ﻿Results

We examined all 1,207 adult Cassidinae specimens deposited in the Insect Collection of the College of Agriculture, Yangtze University, and identified them into 53 species, 13 genera, and 6 tribes. Among them, 14 species are newly recorded in Hubei Province and five in Chongqing. Additionally, *Callispa almora* Maulik, 1923 is recorded for the first time in Zhejiang Province, *Platypria aliena* Chen & Sun, 1962 in Guangxi Zhuang Autonomous Region, and *Cassida rubiginosa* Müller, 1776 in Sichuan.

### ﻿Tribe Aspidomorphini Chapuis, 1875

**Genus *Aspidomorpha* Hope, 1840**

#### Aspidomorpha (s. str.) difformis (Motschulsky, 1860)

Fig. 1

**Material examined. Chongqing**: MBV, 22 VII 2024, 1,413.3 m a.s.l., Liang Zhang, Jialing Chen, Wenbo Liang & Zixiang Wang leg. [1 spec., A-316]; **Hubei**: CLPT, 15 VII 2008, Bo Zhan leg. [1 spec., A-265]; CLPT, 15 VII 2008, Hongyan Liu leg. [1 spec., A-280]; CLPT, 16 VII 2008, Hongyan Liu leg. [1 spec., A-281]; DLLFF, 23 VII 2010, Hongyu Chen leg. [1 spec., A-267]; DLLFF, 25 VII 2010, Xiaoqin Liu leg. [1 spec., A-268]; HH, 16 VII 2002, Daochun Hu leg. [1 spec., A-261]; HH, 17 VII 2002, Caihua Shi leg. [1 spec., A-262]; HH, 18 VII 2002, Caihua Shi leg. [1 spec., A-263]; HH, 16 VII 2002, Yan Yang leg. [1 spec., A-272]; HH, 16 VII 2002, Hongmei Zhang leg. [1 spec., A-273]; HH, 21 VII 2002, Mingsheng Chen leg. [1 spec., A-274]; HLT, 14 VII 2004, Yiping Zou leg. [1 spec., A-271]; LMHV, 26 VI 2010, Wei Li leg. [1 spec., A-266]; LMHV, 25 VI 2010, Guanglin Xie leg. [1 spec., A-269]; LMHV, 27 VI 2010, Guanglin Xie leg. [1 spec., A-270]; LWCV, 3 VII 2015, Ni Cai leg. [2 spec., A-282, A-283]; LWCV, 5 VII 2015, Ni Cai leg. [1 spec., A-284]; WCV, 4 VII 2015, Qi Zhang leg. [1 spec., A-285]; LWCV, 4 VII 2015, Rong Hu leg. [1 spec., A-286]; MYT, 10 VIII 2004, Zengming Zhong leg. [1 spec., A-260]; QGPV, 11 VII 2014, Yuekun Ma leg. [1 spec., A-264]; QGPV, 11 VII 2014, Yao Zuo leg. [1 spec., A-276]; QGPV, 11 VII 2014, Yin Luo leg. [1 spec., A-277]; QGPV, 11 VII 2014, Xingyu Wang leg. [1 spec., A-278]; QGPV, 6 VII 2017, Dong Chen leg. [1 spec., A-279]; RHPT, VII–XI 2013, Shiqi Li leg. [1 spec., A-287]; WJV, 12 VII 2009, Wenjie Sun leg. [1 spec., A-275]; **Sichuan**: FPV, 26 VII 2024, 1,717.2 m a.s.l., 32.686682°N, 104.076656°E, Chaokun Yang leg. [3 spec., A-309–A-311]; SG, 22 VII 2024, 1,783.1 m a.s.l., 32.685896°N, 104.058208°E, Chaokun Yang leg. [1 spec., A-308].

**Figures 1–9.**
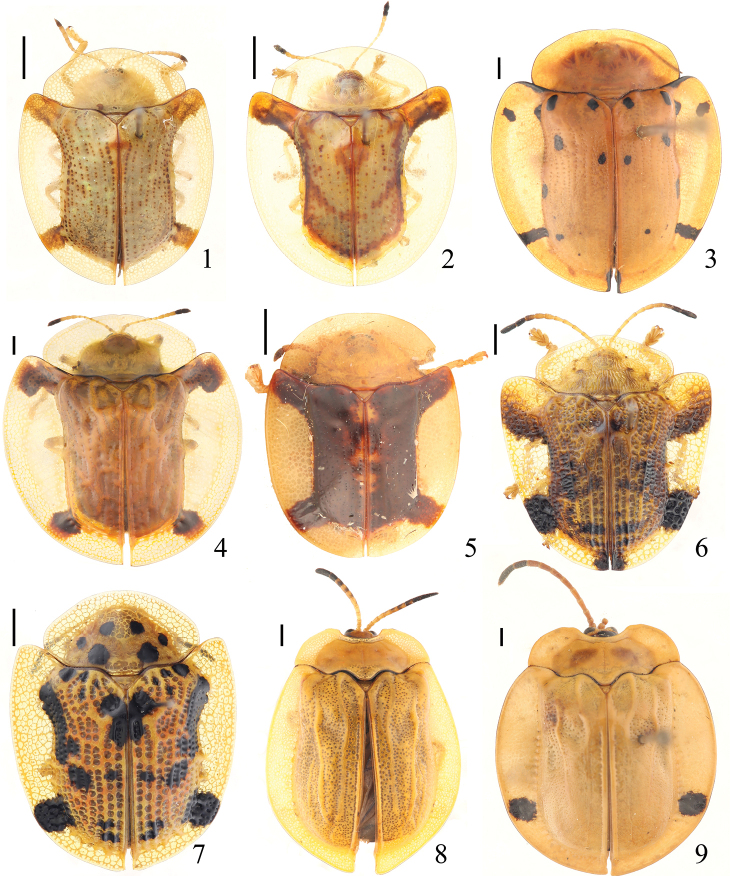
Specimens of Cassidinae deposited in the Insect Collection of the College of Agriculture, Yangtze University **1**Aspidomorpha (s. str.) difformis (Motschulsky, 1860) **2**Aspidomorpha (s. str.) furcata (Thunberg, 1789) **3**Aspidomorpha (s. str.) miliaris (Fabricius, 1775) **4**Aspidomorpha (s. str.) sanctaecrucis (Fabricius, 1792) **5**Aspidomorpha (s. str.) transparipennis (Motschulsky, 1860) **6**Laccoptera (Laccopteroidea) nepalensis Boheman, 1855 **7**Laccoptera (Sindiola) hospita Boheman, 1855 **8***Basiprionota bisignata* (Boheman, 1862) **9***Basiprionota chinensis* (Fabricius, 1798). Scale bars: 1 mm.

**Distribution.** China (Beijing, Chongqing*, Fujian, Gansu, Guizhou, Hebei, Heilongjiang, Hubei, Hunan, Jilin, Liaoning, Shaanxi, Sichuan, Taiwan, Zhejiang); Japan; North Korea; Russia (Far East).

#### ﻿Aspidomorpha (s. str.) furcata (Thunberg, 1789)

Fig. 2

**Material examined. Guangxi**: BNV, 12 VIII 2024, Guanglin Xie leg. [3 spec., A-298–A-300]; SLT, 30 VII 2024, 363 m a.s.l., 24.88°N, 107.97°E, Guanglin Xie leg. [3 spec., A-295–A-297].

**Distribution.** China (Fujian, Guangdong, Guangxi, Guizhou, Hainan, Hunan, Jiangsu, Jiangxi, Macao, Shanghai, Sichuan, Taiwan, Yunnan, Zhejiang); Bhutan; Cambodia; India; Indonesia; Japan; Laos; Malaysia; Myanmar; Nepal; Sri Lanka; Thailand; Vietnam.

#### ﻿Aspidomorpha (s. str.) miliaris (Fabricius, 1775)

Fig. 3

**Material examined. Hainan**: JFL, 2 VI 2010, Wei Li leg. [1 spec., A-256]; JFL, 5 VI 2010, Wei Li leg. [1 spec., A-257].

**Distribution.** China (Guangdong, Guangxi, Hainan, Hong Kong, Taiwan, Yunnan); Bangladesh; India; Indonesia; Japan; Laos; Malaysia; Myanmar; Nepal; New Guinea; Pakistan; Philippines; Thailand; Vietnam.

#### Aspidomorpha (s. str.) sanctaecrucis (Fabricius, 1792)

Fig. 4

**Material examined. Guangxi**: BNV, 12 VIII 2024, Guanglin Xie leg. [3 spec., A-305–A-307]; SLT, 30 VII 2024, 363 m a.s.l., 24.88°N, 107.97°E, Guanglin Xie leg. [1 spec., A-301]; YG, 8 VIII 2024, 389 m a.s.l., 24.81°N, 106.19°E, Guanglin Xie leg. [3 spec., A-302–A-304]; **Guizhou**: CMC, 19 VIII 2024, Chaokun Yang leg. [1 spec., A-312].

**Distribution.** China (Fujian, Guangdong, Guangxi, Hainan, Hunan, Sichuan, Yunnan); America?; Bangladesh; Bhutan; Cambodia; India; Indonesia; Laos; Malaysia; Myanmar; Nepal; Philippines; Pakistan; Sri Lanka; Thailand; Vietnam.

#### Aspidomorpha (s. str.) transparipennis (Motschulsky, 1860)

Fig. 5

**Material examined. Hubei**: WJV, 15 VII 2009, 705.6 m a.s.l., Qing Xiong leg. [1 spec., A-259]; MPT, 13 VII 2009, Yongchao Tao leg. [1 spec., A-258].

**Distribution.** China (Beijing, Hebei, Heilongjiang, Hubei*, Jilin, Liaoning, Shandong); Japan; North Korea; Russia (Far East).

### ﻿Genus *Laccoptera* Boheman, 1855

#### ﻿Laccoptera (Laccopteroidea) nepalensis Boheman, 1855

Fig. 6

**Material examined. Guangxi**: DPV, 7 VIII 2024, Guanglin Xie leg. [2 spec., A-288, A-289]; BNV: 12 VIII 2024, Guanglin Xie leg. [5 spec., A-290–A-294]; **Guizhou**: HTT, 17 VIII 2024, Chaokun Yang & Wenbo Liang leg. [2 spec., A-314, A-315]; NPT 18 VIII 2024, Chaokun Yang & Wenbo Liang leg [3 spec., A-317–A-319]; **Hubei**: BDC, 15 VII 2008, 800 m a.s.l., Shaochuan Wang leg. [1 spec., A-241]; BJMV, 16 VII 2003, Guanglin Xie leg. [1 spec., A-168]; CLPT, 16 VII 2008, Li Jiang leg. [1 spec., A-139]; CLPT, 17 VII 2008, Congxi Bi leg. [1 spec., A-217]; DLLFF, 23 VII 2010, Peng Cheng leg. [4 spec., A-092–A-094, A-098]; DLLFF, 22 VII 2010, Hongyu Chen leg. [1 spec., A-095]; DLLFF, 26 VII 2010, Suting Wang leg. [1 spec., A-096]; DLLFF, 25 VII 2010, Yanfei Huang leg. [1 spec., A-097]; DLLFF, 26 VII 2010, Quanqing Feng leg. [1 spec., A-099]; DLLFF, 23 VII 2010, Zhiqiang Wang leg. [1 spec., A-100]; DLLFF, 22 VII 2010, Long Wang leg. [1 spec., A-101]; DLLFF, 24 VII 2010, Min Tang leg. [2 spec., A-102, A-103]; DLLFF, 23 VII 2010, Jun Wang leg. [1 spec., A-104]; DLLFF, 3 VII 2017, Huihui Xu leg. [1 spec., A-106]; DLLFF, 2 VII 2017, Huihui Xu leg. [1 spec., A-107]; HH, 14 VII 2002, Hongmei Zhang leg. [1 spec., A-050]; HH, 18 VII 2002, Caihua Shi leg. [1 spec., A-169]; HH, 30 IV 2004, 900–1,100 m a.s.l., Guanglin Xie leg. [1 spec., A-219]; HH, 29 IV 2004, 900–1,100 m a.s.l., Guanglin Xie leg. [1 spec., A-227]; HH, 29 IV 2004, 900–1,100 m a.s.l., Wenkai Wang leg. [1 spec., A-238]; HHT, 16 VII 2009, Guanwei Deng leg. [1 spec., A-058]; HHT, 15 VII 2009, Peng Xu leg. [1 spec., A-062]; HHT, 14 VII 2009, Lijie Yu leg. [2 spec., A-063, A-083]; HHT, 13 VII 2009, Guanwei Deng leg. [1 spec., A-065]; HHT, 14 VII 2009, Yajing Xu leg. [2 spec., A-068, A-090]; HHT, 14 VII 2009, Jing Li leg. [3 spec., A-072, A-080, A-091]; HHT, 14 VII 2009, Zhongyi He leg. [1 spec., A-073]; HHT, 16 VII 2009, Zhongyi He leg. [1 spec., A-075]; HHT, 15 VII 2009, Yun Dong leg. [1 spec., A-076]; HHT, 13 VII 2009, Zebo Liang leg. [1 spec., A-077]; HHT, 14 VII 2009, Guanwei Deng leg. [1 spec., A-079]; HHT, 16 VII 2009, Xing Hu leg. [1 spec., A-081]; HHT, 13 VII 2009, Yun Dong leg. [2 spec., A-084, A-088]; HHT, 16 VII 2009, Zebo Zhang leg. [1 spec., A-111]; HHT, 15 VII 2009, Wei Yang leg. [1 spec., A-185]; HHT, 13 VII 2009, Xing Hu leg. [1 spec., A-188]; HLT, 13 VIII 2004, 1,000 m a.s.l., Bi He leg. [1 spec., A-017]; HLT, 15 VIII 2004, 1,000 m a.s.l., Huan Pan leg. [1 spec., A-220]; HLT, 16 VIII 2004, 1,000 m a.s.l., Diwu Cheng leg. [1 spec., A-228]; HLT, 16 VIII 2004, 1,000 m a.s.l., Yiping Zou leg. [1 spec., A-232]; HLT, 14 VII 2004, 1,000 m a.s.l., Shulian Cao leg. [1 spec., A-239]; HMCV, 8 VII 2011, 1,000 m a.s.l., Lihua Ye leg. [1 spec., A-183]; HMCV, 10 VII 2011, 1,000 m a.s.l., Qiyang Huang leg. [1 spec., A-191]; JPV, 10 VII 2008, Qingxiu Zhang leg. [1 spec., A-178]; LLT, 11 VII 2007, Yanli Wang leg. [1 spec., A-021]; LLT, 2 VII 2007, Hu Sun leg. [1 spec., A-037]; LLT, 10 VII 2007, Guangkai Yao leg. [1 spec., A-048]; LLT, 3 VII 2007, Yan Xu leg. [1 spec., A-192]; LLT, 12 VII 2007, Xiaoxiong Liu leg. [1 spec., A-193]; LLT, 13 VII 2007, Hongguan Ma leg. [1 spec., A-213]; LLT, 9 VII 2007, Tao Zhang leg. [1 spec., A-218]; LLT, 10 VII 2007, Wei Wang leg. [1 spec., A-224]; LLT, 9 VII 2007, Qiang Lei leg. [1 spec., A-225]; LLT, 12 VII 2007, Zaibo Huang leg. [1 spec., A-226]; LLT, 10 VII 2007, Rong Xu leg. [1 spec., A-230]; LLT, 10 VII 2007, Xiaoxiong Liu leg. [1 spec., A-242]; LMHV, 27 VI 2010, Wei Li leg. [1 spec., A-020]; LPT, 11 VII 2014, 900 m a.s.l., Bin Wang leg. [1 spec., A-170]; LPT, 11 VII 2014, 900 m a.s.l., Yang Zhang leg. [1 spec., A-171]; LWCV, 4 VII 2015, Xiaobin Zheng leg. [1 spec., A-007]; LWCV, 2 VII 2015, Zhimin Fang leg. [1 spec., A-008]; LWCV, 2 VII 2015, Xiaobin Zheng leg. [1 spec., A-009]; LWCV, 3 VII 2015, Jiajun He leg. [1 spec., A-010]; LWCV, 2 VII 2015, Xiaolong Liu leg. [1 spec., A-011]; LWCV, 5 VII 2015, Rong Hu leg. [1 spec., A-112]; LWCV, 2 VII 2015, Peizhao Dai leg. [1 spec., A-113]; LWCV, 3 VII 2015, Rong Hu leg. [3 spec., A-114–A-116]; LWCV, 5 VII 2015, Qi Zhang leg. [1 spec., A-117]; LWCV, 3 VII 2015, Liu Yu leg. [1 spec., A-118]; LWCV, 4 VII 2015, Yan Ru leg. [1 spec., A-119]; LWCV, 3 VII 2015, Jing Cai leg. [1 spec., A-120]; LWCV, 4 VII 2015, Qi Zhang leg. [1 spec., A-121]; LWCV, 3 VII 2015, Liu Yu leg. [1 spec., A-122]; LWCV, 4 VII 2015, Rong Hu leg. [1 spec., A-123]; LWCV, 4 VII 2015, Jianbin Lv leg. [1 spec., A-124]; LWCV, 4 VII 2015, Xiaoling Li leg. [1 spec., A-125]; LWCV, 6 VII 2015, Xiaoling Li leg. [1 spec., A-126]; LWCV, 6 VII 2015, Ru Yan leg. [1 spec., A-127]; LWCV, 4 VII 2015, Yu Chen leg. [1 spec., A-128]; MPT, 16 VII 2009, Guanglin Xie leg. [2 spec., A-051, A-060]; MPT, 15 VII 2009, Beiyi Guo leg. [1 spec., A-052]; MPT, 13 VII 2009, Jian Zhu leg. [2 spec., A-056, A-071]; MPT, 13 VII 2009, Xiaoxiao Yang leg. [2 spec., A-057, A-069]; MPT, 14 VII 2009, Guanglin Xie leg. [1 spec., A-059]; MPT, 14 VII 2009, Qing Xiong leg. [1 spec., A-061]; MPT, 13 VII 2009, Jiling Liu leg. [1 spec., A-064]; MPT, 12 VII 2009, Guanglin Xie leg. [4 spec., A-066, A-082, A-085, A-086]; MPT, 14 VII 2009, Xiaoxiao Yang leg. [1 spec., A-067]; MPT, 16 VII 2009, Beiyi Guo leg. [1 spec., A-070]; MPT, 13 VII 2009, Yu Tian leg. [1 spec., A-074]; MPT, 14 VII 2009, Jian Zhu leg. [1 spec., A-078]; MPT, 16 VII 2009, Wenjie Yang leg. [1 spec., A-089]; MPT, 16 VII 2009, Xingyu Liu leg. [1 spec., A-108]; MPT, 15 VII 2009, Zijie Zhang leg. [1 spec., A-109]; MPT, 14 VII 2009, Zijie Zhang leg. [1 spec., A-110]; MPT, 17 VII 2008, Jianguo Ke leg. [1 spec., A-129]; MPT, 16 VII 2009, Yanli Tan leg. [1 spec., A-141]; MPT, 15 VII 2009, Yihua Wan leg. [1 spec., A-142]; MPT, 15 VII 2009, Fei Bao leg. [1 spec., A-143]; MPT, 16 VII 2009, Fei Bao leg. [1 spec., A-144]; MPT, 15 VII 2009, Xuemei Sun leg. [1 spec., A-145]; MPT, 14 VII 2009, Fei Bao leg. [1 spec., A-146]; MPT, 16 VII 2009, Yang Hu leg. [1 spec., A-148]; MPT, 14 VII 2009, Yang Hu leg. [1 spec., A-149]; MPT, 15 VII 2009, Yanwei Li leg. [1 spec., A-150]; MPT, 14 VII 2009, Yanli Tan leg. [1 spec., A-151]; MPT, 14 VII 2009, Zheng Wei leg. [3 spec., A-152–A-154]; MPT, 15 VII 2009, Zijie Zhang leg. [1 spec., A-155]; MPT, 13 VII 2009, Xin Huang leg. [1 spec., A-156]; MPT, 13 VII 2009, Lei Zhou leg. [1 spec., A-158]; MPT, 16 VII 2009, Xiaozhen Lu leg. [2 spec., A-159, A-160]; MPT, 15 VII 2009, Yanwei Li leg. [1 spec., A-162]; MPT, 16 VII 2009, Lei Zhou leg. [1 spec., A-163]; MPT, 12 VII 2009, Yanli Tan leg. [1 spec., A-164]; MPT, 13 VII 2009, Lu Chen leg. [1 spec., A-165]; NJHT, 14 VII 2008, Xiang An leg. [1 spec., A-016]; NJHT, 13 VII 2008, Xianhui Zhang leg. [1 spec., A-038]; NJHT, 16 VII 2008, Huilei Zhang leg. [1 spec., A-130]; NJHT, 14 VII 2008, Cuomu Deji leg. [1 spec., A-131]; NJHT, 14 VII 2008, Tao Yan leg. [1 spec., A-204]; NMPV, 11 VII 2005, Mincheng Peng leg. [1 spec., A-022]; NMPV, 14 VII 2005, Huan Liu leg. [1 spec., A-033]; NMPV, 11 VII 2005, Fan Jiang leg. [1 spec., A-201]; QGPV, 2–6 VII 2023, Xiaodie Chen & Guoyi Yang leg. [1 spec., A-001]; QGPV, 29 VI–3 VII 2022, Shenghong Wang & Junjie He leg. [1 spec., A-002]; QGPV, 29 VI–3 VII 2022, Xiang Ke & Jinhou Zhang leg. [1 spec., A-003]; QGPV, 29 VI–3 VII 2022, Shunyang Tan & Jinting Che leg. [1 spec., A-005]; QGPV, 29 VI–3 VII 2022, Jianling Song & Yi Yuan leg. [1 spec., A-006]; QGPV, VII–XI 2013, Mengxiu Cai leg. [1 spec., A-105]; QGPV, 5 VII 2017, Chuan Qin leg. [1 spec., A-133]; QGPV, 6 VII 2017, Zhaoxiang Wang leg. [1 spec., A-134]; QGPV, 6 VII 2017, Zhehui Zhang leg. [1 spec., A-135]; QGPV, 11 VII 2014, Chen Chen leg. [1 spec., A-172]; QGPV, 11 VII 2014, Wenjing Song leg. [1 spec., A-173]; QGPV, 11 VII 2014, Huan Xu leg. [1 spec., A-174]; QGPV, 11 VII 2014, Xia Zhao leg. [1 spec., A-175]; QGPV, 11 VII 2014, Hongyi Xu leg. [1 spec., A-176]; QGPV, 11 VII 2014, Shasha Jiao leg. [1 spec., A-177]; QGPV, 13–17 VII 2018, Li Mao leg. [1 spec., A-179]; QGPV, VII–XI 2013, Junlong Gao leg. [1 spec., A-258]; QJPV, 5 VII 2015, Xiaoqiao Wen leg. [1 spec., A-136]; QJPV, 3 VII 2015, Yuan Lin leg. [1 spec., A-137]; QJPV, 6 VII 2015, Wei Liu leg. [1 spec., A-138]; QJPV, 5 VII 2015, Wei Liu leg. [1 spec., A-140]; QXT, 11 VII 2017, 864 m a.s.l., 32.03333°N, 109.69389°E, Zhixiong Zhou leg. [1 spec., A-004]; QXT, 7 VI 1982, Zhenjun Wei leg. [1 spec., A-013]; RHPT, 8 VII 2011, 600 m a.s.l., Yangchun Wu leg. [1 spec., A-180]; RHPT, 8 VII 2011, 600 m a.s.l., Qian Chen leg. [1 spec., A-181]; RHPT, 8 VII 2011, 600 m a.s.l., Meirong Liang leg. [1 spec., A-182]; RHPT, 8 VII 2011, 600 m a.s.l., Xinyu Pan leg. [1 spec., A-184]; RHPT, 9 VII 2011, 600 m a.s.l., Junxue Li leg. [1 spec., A-186]; RHPT, 8 VII 2011, 600 m a.s.l., Sun Liang leg. [1 spec., A-187]; RHPT, 9 VII 2011, 600 m a.s.l., Yuanxiao Chen leg. [1 spec., A-189]; RHPT, 9 VII 2011, 600 m a.s.l., Cuiyu Lei leg. [1 spec., A-190]; RHPT, VII–XI 2013, Hongyan Li leg. [3 spec., A-244–A-246]; SBT, 20 VII 2003, 900–1,300 m a.s.l., Junkun Shao leg. [1 spec., A-035]; SBT, 19 VII 2003, 900–1,300 m a.s.l., Junkun Shao leg. [1 spec., A-036]; SBT, 18 VII 2003, Sheng Wang leg. [1 spec., A-167]; SBT, 15 VII 2003, Zhigang Yang leg. [1 spec., A-215]; SDS, 14 VII 2005, Man Luo leg. [1 spec., A-018]; SDS, 15 VII 2005, Lijun Ren leg. [1 spec., A-019]; SDS, 15 VII 2005, Hui Chi leg. [1 spec., A-030]; SDS, 13 VII 2005, Manli Wei leg. [1 spec., A-042]; SDS, 15 VII 2005, Bo Shi leg. [1 spec., A-202]; SDS, 11 VII 2005, Yuan Luo leg. [1 spec., A-210]; SDS, 15 VII 2005, Ansuo Dai leg. [1 spec., A-222]; SQPV, 18 VIII 2019, Jinyu Hu leg. [1 spec., A-166]; SQPV, 1–6 VII 2023, Zhijun Wang & Xueqing Hu leg. [1 spec., A-259]; SS, 16 VII 2008, Niu Liu leg [1 spec., A-025]; SS, 14 VII 2008, Shuangfeng Cheng leg. [1 spec., A-055]; SS, 16 VII 2008, Yongtian Lan leg. [1 spec., A-132]; SYST, 12 VII 2004, 1,000 m a.s.l., Yongjiao Wang leg. [1 spec., A-034]; SYST, 12 VII 2004, 1,000 m a.s.l., Ting Liu leg. [1 spec., A-203]; SYST, 12 VII 2004, 1,000 m a.s.l., Haibing Huang leg. [1 spec., A-237]; TYT, 10 VII 2017, 659 m a.s.l., 31.77639°N, 109.92639°E, Xin Liu leg. [1 spec., A-255]; TZS, 12 VII 2005, Xia Wang leg. [1 spec., A-209]; TZS, 12 VII 2005, Xia Wang leg. [1 spec., A-233]; TZS, 11 VII 2005, Yan Chen leg. [1 spec., A-240]; TZS, 11 VII 2005, Xing Ming leg. [1 spec., A-256]; TZS, 14 VII 2005, Qian Wang leg. [1 spec., A-257]; WDHT, 14 VII 2004, 1,000 m a.s.l., Jingping Cai leg. [2 spec., A-235, A-236]; WJV, 12 VII 2009, Guoqiang Chen leg. [1 spec., A-014]; WJV, 12 VII 2009, Ang Lv leg. [1 spec., A-087]; WJV, 14 VII 2009, Liming Liu leg. [1 spec., A-147]; WJV, 13 VII 2009, Yongchao Tao, [1 spec., A-157]; WJV, 13 VII 2009, Guangran Chen leg. [1 spec., A-161]; XBPT, 13 VII 2004, 1,000 m a.s.l., Chengsheng Liu leg. [1 spec., A-028]; XBPT, 14 VII 2004, 1,000 m, Hai Huang leg. [1 spec., A-031]; XBPT, 12 VII 2004, 1,000 m a.s.l., Lei Chen leg. [1 spec., A-040]; XBPT, 15 VII 2004, 1,000 m a.s.l., Jing Li leg. [1 spec., A-044]; XBPT, 10 VII 2004, Chengsheng Liu leg. [1 spec., A-195]; XBPT, 10 VIII 2004, Lei Chen leg. [1 spec., A-221]; XBPT, 11 VIII 2004, 1,000 m a.s.l., Bin Yan leg. [1 spec., A-229]; XBT, 9 VII 2017, 1,061 m a.s.l., 31.59163°N, 109.8169°E, Xin Liu leg. [2 spec., A-253, A-254]; XST, 11 VII 2007, Shengchao Zhang leg. [1 spec., A-024]; XST, 11 VII 2007, Yi Du leg. [1 spec., A-053]; XST, 10 VII 2007, Hanyong Yang leg. [1 spec., A-054]; XST, 10 VII 2007, Biao Yang leg. [1 spec., A-194]; XST, 11 VII 2007, Shengchao Zhang leg. [2 spec., A-197, A-198]; XST, 12 VII 2005, Hong Tian leg. [1 spec., A-200]; XST, 11 VII 2007, Fuxiang Xiang leg. [1 spec., A-211]; XST, 12 VII 2007, Hong Tian leg. [1 spec., A-212]; XST, 12 VII 2007, Xiaojuan Wu leg. [1 spec., A-216]; XST, 9 VII 2007, Shengchao Zhang leg. [1 spec., A-223]; XST, 11 VII 2007, Shengchao Zhang leg. [1 spec., A-243]; YGT, 17 VII 2008, Lizhi Huo leg. [1 spec., A-023]; YGT, 16 VII 2008, Chunmei Xiang leg. [1 spec., A-027]; YGT, 15 VII 2008, Tengfang Lan leg. [1 spec., A-043]; YGT, 16 VII 2008, Lulu Zheng leg. [1 spec., A-207]; YJFF, 10 VII 2007, Xiao Bai leg. [1 spec., A-231]; YSGT, 18 VII 2004, Dingfeng Tan leg. [1 spec., A-234]; ZGW, 14 VII 2005, Kuangfei Xu leg. [1 spec., A-026]; ZGW, 15 VII 2005, Shuibing Lao leg. [2 spec., A-029, A-047]; ZGW, 11 VII 2005, Xiangyuan Yang leg. [1 spec., A-039]; ZGW, 13 VII 2005, Chengzhi Wu leg. [1 spec., A-041]; ZGW, 11 VII 2005, Lei Li leg. [1 spec., A-045]; ZGW, 12 VII 2005, Kuangfei Xu leg. [1 spec., A-046]; ZGW, 12 VII 2005, Chengzhi Wu leg. [1 spec., A-196]; ZGW, 12 VII 2005, Shuibing Lao leg. [1 spec., A-199]; ZGW, 13 VII 2005, Chengzhi Wu leg. [2 spec., A-205, A-214]; ZGW, 12 VII 2005, Chengzhi Wu leg. [1 spec., A-206]; ZGW, 11 VII 2005, Jiaqing Deng leg. [1 spec., A-208]; **Zhejiang**: FYS 28 VII 2007, Liangkui Tan leg. [2 spec., A-032, A-049].

There is no collection information for specimens A-012, A-015, and A-247 to A-252.

**Distribution.** China (Fujian, Guangdong, Guangxi, Guizhou, Hainan, Hubei, Hunan, Jiangsu, Jiangxi, Liaoning, Sichuan, Taiwan, Yunnan, Zhejiang); India; Indonesia; Japan; Laos; Malaysia; Myanmar; Nepal; Pakistan; Singapore; Thailand; Vietnam.

#### ﻿Laccoptera (Sindiola) hospita Boheman, 1855

Fig. 7

**Material examined. Guangxi**: JG, 26 VII 2024, 274 m a.s.l., 24.81°N, 107.97°E, Guanglin Xie leg. [1 spec., A-307].

**Distribution.** China (Guangdong, Guangxi, Guizhou, Hainan, Sichuan, Yunnan); Cambodia; Laos; Thailand; Vietnam.

### ﻿Tribe Basiprionotini Hincks, 1952

**Genus *Basiprionota* Chevrolat, 1836**

#### ﻿*Basiprionota bisignata* (Boheman, 1862)

Fig. 8

**Material examined. Guizhou**: HTT, 17 VIII 2024, Chaokun Yang leg. [1 spec., B-076]; HTW, 21 VIII 2024, Chaokun Yang leg. [2 spec., B-077, B-078]; TLV, 22 VIII 2024, Chaokun Yang leg. [1 spec., B-079]; **Hubei**: DLLFF, 23 VII 2010, Quanqing Feng leg. [1 spec., B-040]; HLT, 16 VII 2004, 1,000 m a.s.l., Shulian Cao leg. [1 spec., B-045]; HLT, 14 VII 2004, 1,000 m a.s.l., Yiping Zou leg. [1 spec., B-046]; HLT, 16 VII 2004, 1,000 m a.s.l., Juan Wei leg. [2 spec., B-047, B-048]; LWCV, 2 VII 2009, Yanguan Chen leg. [1 spec., B-037]; MYT, 13 VII 2004, 1,300 m a.s.l., Jun Huang leg. [1 spec., B-055]; NYT, 15 VII 2004, 1,000 m a.s.l., Hongni Cao leg. [1 spec., B-043]; QGPV, 2–7 VII 2023, Yaqi Wan & Yuhong Wu leg. [2 spec., B-049, B-050]; QGPV, 2–7 VII 2023, Chang Liu & Longxin Ai leg. [4 spec., B-051–B-054]; SQPV, 1–6 VII 2023, Liqing Yuan & Qian Shui leg. [1 spec., B-042]; WJV: 17 VII 2009, Yihao Lou leg. [1 spec., B-038]; WJV, 14 VII 2009, Jianming Miao leg. [1 spec., B-039]; XPV, 20 VI 2008, 1,070 m a.s.l., Guanglin Xie leg. [1 spec., B-044]; XRX, 25 VI 2009, Yu Tian leg. [1 spec., B-041].

**Distribution.** China (Beijing, Gansu, Guangdong, Guangxi, Guizhou, Hebei, Henan, Hubei, Hunan, Jiangsu, Jiangxi, Liaoning, Shanxi, Shandong, Shaanxi, Yunnan, Zhejiang); Malaysia; Thailand; Vietnam.

#### ﻿*Basiprionota chinensis* (Fabricius, 1798)

Fig. 9

**Material examined. Guangxi**: LWV, 12 VIII 2024, Guanglin Xie leg. [1 spec., B-080]; YG, 17 IV 2024, 470 m a.s.l., 24.7979°N, 106.2125°E, Guanglin Xie leg. [1 spec., B-061]; **Guizhou**: HTT, 17 VIII 2024, Chaokun Yang leg. [1 spec., B-074]; NPT, 18 VIII 2024, Chaokun Yang leg. [1 spec., B-075]; **Hubei**: DLLFF, 24 VII 2010, Zuli Yang leg. [1 spec., B-056]; DLLFF, 25 VII 2010, Jiawen Wang leg. [1 spec., B-057]; DLLFF, 26 VII 2010, Rongrong Bai leg. [1 spec., B-058]; DLLFF, 2 V 2010, Jun Zhou leg. [1 spec., B-059].

**Distribution.** China (Fujian, Guangdong, Guangxi, Guizhou, Hainan, Hong Kong, Hubei, Hunan, Jiangsu, Jiangxi, Shaanxi, Shanghai, Sichuan, Zhejiang); Philippines; Vietnam.

#### ﻿*Basiprionota gressitti* Medvedev, 1957

Fig. 10

**Material examined. Hubei**: HLT, 16 VII 2004, 1,000 m a.s.l., Juan Wei leg [1 spec., B-60].

**Distribution.** China (Hubei*, Sichuan).

#### ﻿*Basiprionota pudica* (Spaeth, 1925)

Fig. 11

**Material examined. Guangxi**: PTV, 20 VII 2024, 765 m a.s.l., 24.03°N, 107.73°E, Guanglin Xie leg. [1 spec., B-062]; SHV, 22 IV 2024, 980 m a.s.l., 24.6184°N, 106.4723°E, Guanglin Xie leg. [1 spec., B-067]; XLV, 25 IV 2024, 760 m a.s.l., 24.5404°N, 107.0673°E, Guanglin Xie leg. [1 spec., B-066]; YG, 8 VIII 2024, 389 m a.s.l., 24.81°N, 106.19°E, Guanglin Xie leg. [3 spec., B-063–B-065]; **Guizhou**: XJT, 5 VI 2007, 630–650 m a.s.l., Guanglin Xie leg. [1 spec., B-036]; **Hubei**: LPT, 11 VII 2014, 900 m a.s.l., Xian Wang leg. [1 spec., B-035].

**Distribution.** China (Guangxi, Guizhou, Hubei, Hunan, Sichuan, Yunnan); India.

#### ﻿*Basiprionota whitei* (Boheman, 1856)

Fig. 12

**Material examined. Guizhou**: CMC, 19 VIII 2024, 699 m a.s.l., Chaokun Yang leg. [5 spec., B-068–B-072]; TLV, 23 VIII 2024, Chaokun Yang leg. [1 spec., B-073].

**Figures 10–16.**
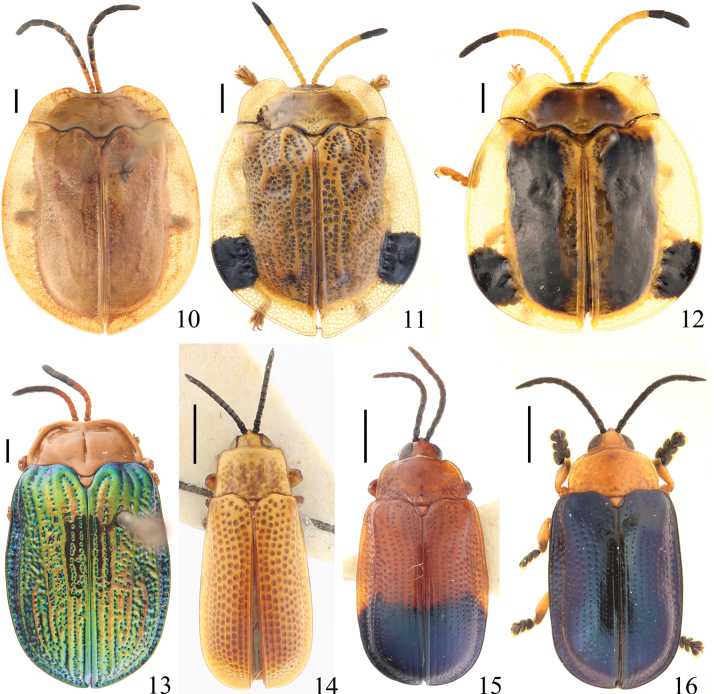
Specimens of Cassidinae deposited in the Insect Collection of the College of Agriculture, Yangtze University **10***Basiprionota gressitti* Medvedev, 1957 **11***Basiprionota pudica* (Spaeth, 1925) **12***Basiprionota whitei* (Boheman, 1856) **13***Craspedonta leayana* (Latreille, 1807) **14***Callispa almora* Maulik, 1923 **15***Callispa dimidiatipennis* Baly, 1858 **16***Callispa donckieri* Pic, 1924. Scale bars: 1 mm.

**Distribution.** China (Anhui, Fujian, Guangdong, Guangxi, Guizhou, Hunan, Jiangsu, Jiangxi, Zhejiang).

### ﻿Genus *Craspedonta* Chevrolat, 1837

#### ﻿*Craspedonta leayana* (Latreille, 1807)

Fig. 13

**Material examined. Hainan**: JFL, 1 VI 2010, Wei Li leg. [3 spec., B-001–B-003]; JFL, 2 VI 2010, Wei Li leg. [9 spec., B-004–B-012]; JFL, 3 VI 2010, Wei Li leg. [16 spec., B-013–B-028]; JFL, 6 VI 2010, Wei Li leg. [6 spec., B-029–B-034].

**Distribution.** China (Hainan, Yunnan); India; Laos; Myanmar; Nepal; Thailand; Vietnam.

### ﻿Tribe Callispini Chapuis, 1875

**Genus *Callispa* Baly, 1858**

#### ﻿*Callispa almora* Maulik, 1923

Fig. 14

**Material examined. Zhejiang**: FYS, 27 VII 2007, Liangkui Tan leg. [1 spec., CL-002].

**Distribution.** China (Fujian, Sichuan, Yunnan; Zhejiang*); India.

#### ﻿*Callispa dimidiatipennis* Baly, 1858

Fig. 15

**Material examined. Hubei**: HH, 2 V 2004, Wenkai Wang leg. [1 spec., CL-001].

**Distribution.** China (Guangxi, Guizhou, Hainan, Hubei*, Hunan, Yunnan); India; Laos; Myanmar; Vietnam.

#### ﻿*Callispa donckieri* Pic, 1924

Fig. 16

**Material examined. Guizhou**: KKS, 11 VII 2024, 28.2345°N, 107.1588°E, Chaokun Yang leg. [2 spec., CL-003, CL-004].

**Distribution.** China (Fujian, Guangxi, Guizhou, Sichuan).

### ﻿Tribe Cassidini Gyllenhal, 1813

**Genus *Cassida* Linnaeus, 1758**

#### ﻿*Cassida australica* (Boheman, 1855)

Fig. 17

**Material examined. Hubei**: BJMV, 18 VII 2003, 900–1,300 m a.s.l., Guanglin Xie leg. [1 spec., CS-319]; LMH, 25 VII 2003, Wei Li leg. [1 spec., CS-322]; WSFF, 18 VII 2003, 1,700–2,000 m a.s.l., Deshou Song leg. [1 spec., CS-320]; WSFF, 19 VII 2003, 900–1,300 m a.s.l., Suran Wu leg. [1 spec., CS-321]; **Sichuan**: FPV, 26 VII 2024, 1,717.2 m a.s.l., 32.686682°N, 104.076656°E, Chaokun Yang leg. [3 spec., CS-630–CS-632]; FPV, 26 VII 2024, 1,694.8 m a.s.l., 32.685468°N, 104.076105°E, Chaokun Yang leg. [4 spec., CS-633–CS-636].

**Distribution.** China (Guizhou, Hubei*, Sichuan, Xizang, Yunnan); India; Laos; Myanmar; Nepal; Thailand; Vietnam.

#### ﻿*Cassida circumdata* Herbst, 1799

Fig. 18

**Material examined. Guangxi**: BAT, 18 VII 2024, 404.6 m a.s.l., 24.118466°N, 107.825591°E, Guanglin Xie leg. [1 spec., CS-612]; BNV, 12 VIII 2024, Guanglin Xie leg. [2 spec., CS-610, CS-611]; **Guizhou**: NPT, 18 VIII 2024, Chaokun Yang leg. [12 spec., CS-657–CS-668]; HTT, 17 VIII 2024, Chaokun Yang leg. [1 spec., CS-669]; **Hubei**: HBAU, 1 VIII 2001, [1 spec., CS-124]; HBAU, 23 VIII 2001, Fansong Zeng leg. [1 spec., CS-148]; HBAU, 25 VIII 2001, Yongsheng Yan leg. [1 spec., CS-149]; HBAU, 25 VIII 2001, Guanjun Cen leg. [1 spec., CS-150]; HBAU, 25 VIII 2001, Qingping Yan leg. [1 spec., CS-151]; HBAU, 25 VIII 2001, Jiabing Fu leg. [1 spec., CS-152]; HBAU, 25 VIII 2001, Min Tu leg. [1 spec., CS-153]; HBAU, 24 VIII 2001, Lifang Zhou leg. [1 spec., CS-154]; HBAU, 25 VIII 2001, Zuqun Liu leg. [2 spec., CS-155, CS-156]; HBAU, 24 VIII 2001, Sha Chen leg. [1 spec., CS-157]; HBAU, 23 VIII 2001, He Huang leg. [1 spec., CS-158]; HBAU, 23 VIII 2001, Dan Chen leg. [1 spec., CS-159]; HBAU, 23 VIII 2001, Maohua Xu leg. [1 spec., CS-160]; HZS, 11 VII 2007, Leizhi Wei leg. [1 spec., CS-119]; JZ, 28 VII 2002, Shuangxi Zhou leg. [1 spec., CS-123]; LLT, 11 VII 2007, Rong Xu leg. [1 spec., CS-122]; MPT, 13 VII 2009, Wenjie Yang leg. [1 spec., CS-117]; MPT, 14 VII 2009, Lei Zhou leg. [1 spec., CS-121]; NMPV, 14 VII 2005, Yan Kong leg. [1 spec., CS-125]; SYPV, 11 VII 2005, Liping Yang leg. [1 spec., CS-118]; SYST, 12 VII 2004, Yongjiao Wang leg. [1 spec., CS-120]; WDHT, 10 VIII 2004, 1,000 m a.s.l., Yagang Bao leg. [1 spec., CS-207]; XST, 11 VII 2007, Biao Yang leg. [1 spec., CS-116].

**Figures 17–25.**
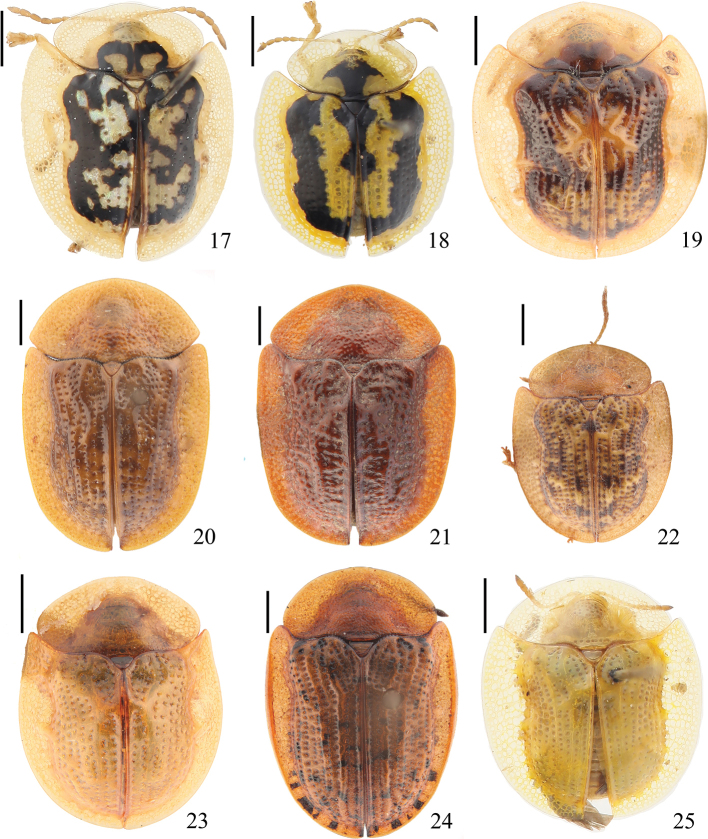
Specimens of Cassidinae deposited in the Insect Collection of the College of Agriculture, Yangtze University **17***Cassida australica* (Boheman, 1855) **18***Cassida circumdata* Herbst, 1799 **19***Cassida expansa* Gressitt, 1952 **20***Cassida fuscorufa* Motschulsky, 1866 **21***Cassida jacobsoni* Spaeth, 1914 **22***Cassida japana* Baly, 1874 **23***Cassida juglans* Gressitt, 1942 **24***Cassida nebulosa* Linnaeus, 1758 **25***Cassida obtusata* Boheman, 1854. Scale bars: 1 mm.

**Distribution.** China (Fujian, Guangdong, Guangxi, Guizhou, Hainan, Hubei, Hunan, Jiangsu, Jiangxi, Sichuan, Taiwan, Yunnan, Zhejiang); Bangladesh; India; Indonesia; Japan; Laos; Malaysia; Nepal; Philippines; Sri Lanka; Thailand; Vietnam.

#### ﻿*Cassida expansa* Gressitt, 1952

Fig. 19

**Material examined. Hubei**: MYT, 12 VIII 2004, 900 m a.s.l., Guijiang Meng leg. [1 spec., CS-208].

**Distribution.** China (Hainan, Hubei*); Vietnam.

#### ﻿*Cassida fuscorufa* Motschulsky, 1866

Fig. 20

**Material examined. Hubei**: DLLFF, 25 VII 2010, Min Tang leg. [1 spec., CS-245]; DLLFF, 23 VII 2010, Jian Zhang leg. [1 spec., CS-271]; DLLFF, 25 VII 2010, Yuting Yang leg. [1 spec., CS-272]; DLLNNR, 7 VII 2011, 1,200 m a.s.l., Hui Dong leg. [1 spec., CS-244]; DLLNNR, 9 VII 2011, 1,200 m a.s.l., Ling Huang leg. [1 spec., CS-268]; DLLNNR, 7 VII 2011, 1,200 m a.s.l., Xiuqiong Li leg. [1 spec., CS-269]; DLLNNR, 11 VII 2011, 1,200 m a.s.l., Xiaoyang Li leg. [1 spec., CS-270]; FXT, 12 VII 2017, 31.91028°N, 109.76444°E, Zhixiong Zhang leg. [2 spec., CS-289, CS-290]; HH, 18 VII 2002, Linling Li leg. [1 spec., CS-247]; HH, 18 VII 2002, Chaowu Liu leg. [1 spec., CS-248]; HH, 15 VII 2002, Shouhe Yu leg. [1 spec., CS-250]; HH, 20 VII 2002, Shouhe Yu leg. [1 spec., CS-252]; HH, 16 VII 2002, Hongmei Zhang leg. [1 spec., CS-254]; HH, 20 VII 2002, Pei Zhong leg. [1 spec., CS-255]; HHT, 13 VII 2009, Guanwei Deng leg. [1 spec., CS-240]; HHT, 15 VII 2017, 417 m a.s.l., 32.10889°N, 109.81444°E, Jiabo Wang leg. [1 spec., CS-241]; HHT, 16 VII 2009, Zhendong Guo leg. [1 spec., CS-279]; HHT, 15 VII 2009, Jing Li leg. [1 spec., CS-291]; HHT, 18 VII 2002, Chaowu Liu leg. [1 spec., CS-599]; HMCV, 8 VII 2011, 1,000 m a.s.l., Xinxin Li leg. [1 spec., CS-259]; JZ, 24 VII 2002, Kaiqing Huang leg. [1 spec., CS-285]; JZ, 20 VII 2002, Shuangxi Zhou leg. [1 spec., CS-286]; JZ, 23 VII 2002, Jun Guo leg. [1 spec., CS-287]; LLT, 13 VII 2007, Tao Zhang leg. [1 spec., CS-263]; LPT, 4 VII 2012, 900 m a.s.l., Ling Li leg. [1 spec., CS-282]; LPT, 1 VII 2012, 900 m a.s.l., Shibo Zhang leg. [1 spec., CS-283]; LWCV, 4 VII 2015, Qi Zhang leg. [1 spec., CS-600]; MYT, 11 VIII 2004, 900 m a.s.l., Guangzhi Liu leg. [1 spec., CS-264]; MYT, 12 VIII 2004, 1,200 m a.s.l., Jingyang Peng leg. [1 spec., CS-265]; NJHT, 16 VII 2008, Junchao Wang leg. [1 spec., CS-274]; NJHT, 13 VII 2008, Tingting Chen leg. [1 spec., CS-275]; NJHT, 16 VII 2008, Zukai Fang leg. [1 spec., CS-293]; QGPV, 8 VII 2016, Weihao Feng leg. [1 spec., CS-284]; QGPV, 8 VII 2017, Dong Chen leg. [1 spec., CS-292]; QGPV, 3 VII 2012, 1,000 m a.s.l., Zhihao Guo leg. [1 spec., CS-294]; SBT, 16 VII 2003, 900–1,300 m a.s.l., Yueqin Xiang leg. [1 spec., CS-242]; SBT, 19 VII 2003, 900–1,300 m a.s.l., Pengfei Quan leg. [1 spec., CS-243]; SBT, 21 VII 2003, 900–1,300 m a.s.l., Junkun Shao leg. [2 spec., CS-276, CS-277]; SBT, 18 VII 2003, 1,200–1,300 m a.s.l., Haibo Chen leg. [1 spec., CS-278]; SDS, 15 VII 2005, Hui Chi leg. [1 spec., CS-260]; SDS, 14 VII 2005, Yuan Luo leg. [1 spec., CS-261]; SDS, 14 VII 2005, Zewen Zhang leg. [1 spec., CS-262]; SYST, 12 VIII 2004, 1,000 m a.s.l., Ting Liu leg. [1 spec., CS-266]; WJV, 13 VII 2009, Xuntan Jiang leg. [1 spec., CS-239]; WJV, 13 VII 2009, Ang Li leg. [1 spec., CS-280]; WJV, 14 VII 2009, Sai Sun leg. [1 spec., CS-281]; XBT, 8 VII 2017, 31.54811°N, 109.85988°E, Xin Liu leg. [1 spec., CS-288]; XST, 10 VII 2007, Shengchao Zhang leg. [1 spec., CS-257]; XST, 12 VII 2007, Fuxiang Xiang leg. [1 spec., CS-258].

**Distribution.** China (Fujian, Gansu, Guangdong, Guangxi, Hainan, Hebei, Henan, Heilongjiang, Hubei, Hunan, Inner Mongolia, Jilin, Jiangsu, Jiangxi, Liaoning, Shaanxi, Shandong, Shanxi, Sichuan, Taiwan, Zhejiang); Japan; North Korea; Russia (Far East).

#### ﻿*Cassida jacobsoni* Spaeth, 1914

Fig. 21

**Material examined. Hubei**: DLLFF, 26 VII 2010, Lingdong Hu leg. [1 spec., CS-273]; DLLNNR, 9 VII 2011, 1,200 m a.s.l., Ling Huang leg. [1 spec., CS-267]; HH, 14 VII 2002, Xinhou Zhang leg. [1 spec., CS-246]; HH, 18 VII 2002, Linjun Hong leg. [1 spec., CS-249]; HH, 15 VII 2002, Shouhe Yu leg. [2 spec., CS-250, CS-251]; HH, 19 VII 2002, Hongmei Zhang leg. [1 spec., CS-253]; HH, 16 VII 2002, Pei Zhong leg. [1 spec., CS-256]; MYT, 11 VIII 2004, 900 m a.s.l., Guangzhi Liu leg. [1 spec., CS-264]; MYT, 12 VIII 2004, 1,200 m a.s.l., Jingyang Peng leg. [1 spec., CS-265].

**Distribution.** China (Fujian, Hubei, Shaanxi, Zhejiang).

#### ﻿*Cassida japana* Baly, 1874

Fig. 22

**Material examined. Guizhou**: KKS, 11 VIII 2024, Chaokun Yang leg. [4 spec., CS-602, CS-654–CS-656]; **Hubei**: CLPT, 14 VII 2008, Baoqiang Gao leg. [1 spec., CS-177]; HBAU, XI 2000, [1 spec., CS-167]; HH, 20 VII 2002, Shouhe Yu leg. [1 spec., CS-163]; HH, 16 VII 2002, Zhengyu Xiang leg. [1 spec., CS-164]; HH, 19 VII 2002, Haifeng Zhang leg. [1 spec., CS-168]; HH, 19 VII 2002, Yumin Yang leg. [1 spec., CS-169]; HH, 24 VII 2002, Xiangqian Hu leg. [1 spec., CS-171]; HH, 18 VII 2002, Shuai Yan leg. [1 spec., CS-185]; HH, 16 VII 2002, Li Wang leg. [1 spec., CS-190]; HH, 21 VII 2002, Na Pan leg. [1 spec., CS-191]; HHT, 16 VII 2009, Fei Bao leg. [1 spec., CS-324]; HLT, 14 VIII 2004, 1,000 m a.s.l., Yiping Zou leg. [1 spec., CS-178]; HLT, 14 VIII 2004, 1,000 m a.s.l., Bi He leg. [1 spec., CS-205]; HLT, 12 VIII 2004, 1,000 m a.s.l., Xiaogui Zhou leg. [1 spec., CS-209]; JJYT, 17 VII 2017, 676 m a.s.l., 32.31419°N, 109.51542°E, Xihuan Zhou leg. [1 spec., CS-201]; JJYT, 17 VII 2017, 577 m a.s.l., 32.33432°N, 109.57813°E, Xin Liu leg. [2 spec., CS-202, CS-203]; JZ: 29 VII 2002, Xiaoyan Wang leg. [1 spec., CS-162]; JZ: Ming Qin leg. [1 spec., CS-180]; LPT, 11 VII 2014, 900 m a.s.l., Guangze Zheng leg. [1 spec., CS-206]; LWCV, 3 VII 2015, Sui Ye leg. [1 spec., CS-192]; LWCV, 6 VII 2015, Sui Ye leg. [1 spec., CS-193]; QGPV, 3 VII 2012, 1,000 m a.s.l., Shengbiao Zhong leg. [1 spec., CS-170]; QXT, 10 VII 2017, 771 m a.s.l., 32.07611°N, 109.66278°E, Jiabo Wang leg. [1 spec., CS-196]; QXT, 11 VII 2017, 952 m a.s.l., 32.03167°N, 109.68667°E, Zhixiong Zhou leg. [1 spec., CS-197]; QXT, 13 VII 2017, 856 m a.s.l., 32.06861°N, 109.66139°E, Panlong Guo leg. [1 spec., CS-198]; RHPT, VII–XI 2013, Bin Chen leg. [1 spec., CS-195]; SBT, 16 VII 2017, 382 m a.s.l., Zhenzhou Xia leg. [1 spec., CS-188]; SDS, 11 VII 2005, Cuntao Yue leg. [1 spec., CS-174]; SDS, 12 VII 2005, Yuan Luo leg. [1 spec., CS-175]; SDS, 12 VII 2005, Zhigang Liang leg. [1 spec., CS-179]; SPT, 18 VII 2017, 882 m a.s.l., 32.28603°N, 109.84147°E, Zhenzhou Xia leg. [1 spec., CS-204]; SS, 14 VII 2008, Wuyun Wang leg. [1 spec., CS-176]; SYST, 12 VIII 2004, 1,000 m a.s.l., Haibing Huang leg. [1 spec., CS-172]; SZPT, 7 VII 2011, 400 m a.s.l., Shaolin Hong leg. [1 spec., CS-189]; SZPT, 10 VII 2011, 400 m a.s.l., Zezhou Wang leg. [1 spec., CS-325]; WJV, 14 VII 2009, Yihao Lou leg. [1 spec., CS-161]; WJV, 13 VII 2009, Chengfei Niu leg. [1 spec., CS-166]; WJV, 16 VII 2009, Wei Zhou leg. [1 spec., CS-173]; WJV, 14 VII 2009, Xuntan Jiang leg. [1 spec., CS-181]; WJV, 16 VII 2009, Guangran Chen leg. [2 spec., CS-183, CS-184]; WJV, 16 VII 2009, Zhizhi Zhong leg. [1 spec., CS-186]; WJV, 13 VII 2009, Shishui Xie leg. [1 spec., CS-194]; WJV, 14 VII 2009, Xiaotao Lei leg. [1 spec., CS-323]; XBT, 5 VII 2017, 1,208 m a.s.l., 31.5641°N, 109.86863°E, Zhenzhou Xia leg. [2 spec., CS-199, CS-200]; XST, 12 VII 2002, Biao Yang leg. [1 spec., CS-165]; ZFT, 16 VII 2017, 485 m a.s.l., Jiabo Wang leg. [1 spec., CS-187]; ZGW, 12 VII 2005, Lei Li leg. [1 spec., CS-182].

**Distribution.** China (Anhui, Fujian, Guangdong, Guangxi, Guizhou, Hubei, Hunan, Jiangsu, Jiangxi, Shanghai, Sichuan, Taiwan, Yunnan, Zhejiang); Japan; Laos; Vietnam.

#### ﻿*Cassida juglans* Gressitt, 1942

Fig. 23

**Material examined. Hubei**: WSFF, 16 VII 2003, 1,700–2,000 m a.s.l., Qinghua Zhou leg. [1 spec., CS-302].

**Distribution.** China (Hubei*, Zhejiang); North Korea.

#### ﻿*Cassida nebulosa* Linnaeus, 1758

Fig. 24

**Material examined. Hubei**: HH, 16 VII 2002, Pei Zhong leg. [1 spec., CS-219]; HH, 17 VII 2002, Linjun Hong leg. [1 spec., CS-220]; HH, 18 VII 2002, Pei Zhong leg. [3 spec., CS-222–CS-224]; HH, 14 VII 2002, Hongmei Zhang leg. [1 spec., CS-225]; HH, 15 VII 2002, Hongmei Zhang leg. [1 spec., CS-226]; HH, 18 VII 2002, Hongmei Zhang leg. [1 spec., CS-227]; HH, 14 VII 2002, Linjun Hong leg. [1 spec., CS-228]; HH, 15 VII 2002, Yangang Zheng leg. [1 spec., CS-229]; HH, 20 VII 2002, Haifeng Zhang leg. [1 spec., CS-230]; HH, 26 VII 2002, Ping Shen leg. [1 spec., CS-231]; HH, 16 VII 2002, Linjun Hong leg. [1 spec., CS-326]; HH, 19 VII 2002, Shuangxi Zhou leg. [1 spec., CS-327]; HPT, 12 VIII 2004, 1,200 m a.s.l., Jiande Mao leg. [1 spec., CS-234]; JZ, 26 VII 2002, Ying Shi leg. [2 spec., CS-218, CS-221]; MYT, 12 VIII 2004, 1,200 m a.s.l., Jun Huang leg. [1 spec., CS-235]; MYT, 13 VIII 2004, 1,300 m a.s.l., Min Lei leg. [1 spec., CS-236]; SDS, 11 VII 2005, Hui Chi leg. [1 spec., CS-237]; SDS, 11 VII 2005, Ansuo Dai leg. [1 spec., CS-238]; SDS, 12 VII 2005, Manli Wei leg. [1 spec., CS-328]; WJV, 16 VII 2009, Wei Zhou leg. [1 spec., CS-232]; WSFF, 18 VII 2003, 1,700–2,000 m a.s.l., Liang Yang leg. [1 spec., CS-233]; WSFF, 16 VII 2003, 1,700–2,000 m a.s.l., Chuang Gui leg. [1 spec., CS-329]; WSFF, 17 VII 2003, 1,700–2,000 m a.s.l., Liangkui Tan leg. [1 spec., CS-330]; WSFF, 17 VII 2003, 1,700–2,000 m a.s.l., Xiaochun Jin leg. [1 spec., CS-331]; WSFF, 19 VII 2003, 1,700–2,000 m a.s.l., Liang Yang leg. [1 spec., CS-332]; WSFF, 19 VII 2003, 1,700–2,000 m a.s.l., Jing Zheng leg. [1 spec., CS-333]; WSFF, 20 VII 2003, 1,700–2,000 m a.s.l., Wenkai Wang leg. [1 spec., CS-334].

**Distribution.** China (Beijing, Gansu, Hebei, Heilongjiang, Hubei, Inner Mongolia, Jiangsu, Jilin, Liaoning, Ningxia, Shaanxi, Shandong, Shanxi, Shanghai, Sichuan, Tianjin, Xinjiang); Japan; North Korea; Russia; Widely distributed in most of Europe.

#### ﻿*Cassida obtusata* Boheman, 1854

Fig. 25

**Material examined. Guangxi**: BNV, 12 VIII 2024, Guanglin Xie leg. [1 spec., CS-624].

**Distribution.** China (Fujian, Guangdong, Guangxi, Hainan, Hong Kong, Taiwan, Yunnan); Cambodia; India; Indonesia; Japan; Laos; Malaysia; Myanmar; Nepal; Philippines; Sri Lanka; Thailand; Vietnam.

#### ﻿*Cassida pallidicollis* Boheman, 1856

Fig. 26

**Material examined. Hubei**: LWCV, 5 VII 2015, Rong Hu leg. [1 spec., CS-301]; MYT, 12 VIII 2004, 900 m a.s.l., Kun Zhang leg. [1 spec., CS-299]; NMP, 11 VII 2005, Ziwu Qin leg. [1 spec., CS-300]; QGPV, 10 VII 2016, Yuanjie Guo leg. [1 spec., CS-297]; WSFF, 17 VII 2003, 1,700–2,000 m a.s.l., Wei Ding leg. [1 spec., CS-295]; WSFF, 17 VII 2003, 1,700–2,000 m a.s.l., Feng Chen leg. [1 spec., CS-296]; XBT, 5 VII 2017, 1,208 m a.s.l., 31.5641°N, 109.86863°E, Zhenzhou Xia leg. [1 spec., CS-298].

**Distribution.** China (Anhui, Guangxi, Hebei, Heilongjiang, Hubei*, Inner Mongolia, Jiangsu, Jilin, Shanxi, Shandong, Zhejiang); Mongolia; North Korea; Russia (Far East).

#### ﻿*Cassida piperata* Hope, 1842

Fig. 27

**Material examined. Hubei**: BDC, 16 VII 2006, Jian Huang leg. [1 spec., CS-590]; DLLFF, 22 VII 2010, Zhiqiang Wang leg. [1 spec., CS-572]; HBAU, VIII 2001, [1 spec., CS-591]; HBAU, II 2001, Yehong Zhang leg. [1 spec., CS-592]; HBAU, XI 2001, Mingliang Wei leg. [1 spec., CS-594]; HH, 14 VII 2002, Taiyun Ji leg. [5 spec., CS-399–CS-404]; HH, 15 VII 2002, Taiyun Ji leg. [4 spec., CS-405–CS-407, CS-424]; HH, 17 VII 2002, Taiyun Ji leg. [4 spec., CS-408–CS-411]; HH, 18 VII 2002, Taiyun Ji leg. [3 spec., CS-412–CS-414]; HH, 19 VII 2002, Taiyun Ji leg. [8 spec., CS-415–CS-422]; HH, 16 VII 2002, Taiyun Ji leg. [1 spec., CS-423]; HH, 19 VII 2002, Mingliang Wei leg. [5 spec., CS-425–CS-429]; HH, 20 VII 2002, Mingliang Wei leg. [7 spec., CS-430–CS-436]; HH, 21 VII 2002, Mingliang Wei leg. [5 spec., CS-437–CS-441]; HH, 17 VII 2002, Zhengyu Xiang leg. [1 spec., CS-442]; HH, 18 VII 2002, Zhengyu Xiang leg. [1 spec., CS-443]; HH, 17 VII 2002, Kaiqing Huang leg. [1 spec., CS-444]; HH, 5 X 2022, Lijun Cai leg. [1 spec., CS-445]; HH, 16 VII 2002, Haibo Chen leg. [1 spec., CS-446]; HH, 15 VII 2002, Yangang Zheng leg. [1 spec., CS-447]; HH, 18 VII 2002, Tianpeng Zhang leg. [1 spec., CS-448]; HH, 14 VII 2002, Fangping Fu leg. [1 spec., CS-449]; HH, 15 VII 2002, Fangping Fu leg. [2 spec., CS-450, CS-451]; HH, 14 VII 2002, Linjun Hong leg. [1 spec., CS-452]; HH, 17 VII 2002, Linjun Hong leg. [1 spec., CS-453]; HH, 18 VII 2002, Linjun Hong leg. [1 spec., CS-454]; HH, 20 VII 2002, Linjun Hong leg. [1 spec., CS-455]; HH, 19 VII 2002, Xiaowei Zhang leg. [2 spec., CS-456, CS-457]; HH, 16 VII 2002, Xiangzhuang Li leg. [1 spec., CS-458]; HH, 20 VII 2002, Xiangzhuang Li leg. [1 spec., CS-459]; HH, 14 VII 2002, Linling Li leg. [1 spec., CS-460]; HH, 18 VII 2002, Linling Li leg. [2 spec., CS-461, CS-462]; HH, 19 VII 2002, Linling Li leg. [1 spec., CS-463]; HH, 20 VII 2002, Linling Li leg. [2 spec., CS-464, CS-465]; HH, 19 VII 2002, Jun Guo leg. [1 spec., CS-466]; HH, 28 VII 2002, Huifang Li leg. [1 spec., CS-467]; HH, 19 VII 2002, Yumin Yang leg. [4 spec., CS-468–CS-471]; HH, 20 VII 2002, Yumin Yang leg. [4 spec., CS-472–CS-475]; HH, 17 VII 2002, Li Wang leg. [1 spec., CS-476]; HH, 18 VII 2002, Li Wang leg. [1 spec., CS-477]; HH, 21 VII 2002, Li Wang leg. [2 spec., CS-478, CS-479]; HH, 14 VII 2002, Shuangxi Zhou leg. [1 spec., CS-480]; HH, 17 VII 2002, Shuangxi Zhou leg. [1 spec., CS-481]; HH, 19 VII 2002, Shuangxi Zhou leg. [5 spec., CS-482–CS-486]; HH, 14 VII 2002, Shouhe Yu leg. [1 spec., CS-487]; HH, 15 VII 2002, Shouhe Yu leg. [1 spec., CS-488]; HH, 21 VII 2002, Shouhe Yu leg. [1 spec., CS-489]; HH, 14 VII 2002, Pei Zhong leg. [1 spec., CS-490]; HH, 15 VII 2002, Pei Zhong leg. [3 spec., CS-491–CS-493]; HH, 16 VII 2002, Pei Zhong leg. [2 spec., CS-494, CS-495]; HH, 17 VII 2002, Pei Zhong leg. [1 spec., CS-496]; HH, 14 VII 2002, Hongmei Zhang leg. [1 spec., CS-497]; HH, 19 VII 2002, Hongmei Zhang leg. [1 spec., CS-498]; HH, 27 VII 2002, Taiyun Ji leg. [1 spec., CS-499]; HH, 16 VII 2002, Linjun Hong leg. [1 spec., CS-500]; HH, 15 VII 2002, Shuibing Lao leg. [4 spec., CS-501–CS-504]; HH, 16 VII 2002, Shuibing Lao leg. [1 spec., CS-505]; HH, 18 VII 2002, Shuibing Lao leg. [4 spec., CS-506–CS-509]; HH, 18 VII 2002, Feiyong Wang leg. [5 spec., CS-515–CS-519]; HH, 19 VII 2002, Feiyong Wang leg. [2 spec., CS-520, CS-521]; HH, 20 VII 2002, Feiyong Wang leg. [5 spec., CS-522–CS-526]; HH, 14 VII 2002, Shuai Yan leg. [5 spec., CS-527–CS-531]; HH, 16 VII 2002, Shuai Yan leg. [5 spec., CS-532–CS-536]; HH, 18 VII 2002, Shuai Yan leg. [4 spec., CS-537–CS-540]; HH, 20 VII 2002, Shuai Yan leg. [4 spec., CS-541–CS-544]; HH, 14 VII 2002, Pengbo Luo leg. [6 spec., CS-545–CS-550]; HH, 15 VII 2002, Pengbo Luo leg. [4 spec., CS-551–CS-554]; HH, 16 VII 2002, Pengbo Luo leg. [5 spec., CS-555–CS-559]; HH, 17 VII 2002, Pengbo Luo leg. [3 spec., CS-560–CS-562]; HH, 19 VII 2002, Pengbo Luo leg. [3 spec., CS-563–CS-565]; HH, 21 VII 2002, Pengbo Luo leg. [5 spec., CS-566–CS-570]; HHT, 13 VII 2009, Jing Li leg. [1 spec., CS-574]; HHT, 16 VII 2009, Jing Li leg. [1 spec., CS-575]; HLT, 13 VIII 2004, 1,000 m a.s.l., Hongli Yin leg. [1 spec., CS-514]; JZ, 28 VII 2002, Shuangxi Zhou leg. [3 spec., CS-335–CS-337]; JZ, 22 VII 2002, Shuangxi Zhou leg. [2 spec., CS-338, CS-339]; JZ, 23 VII 2002, Shuangxi Zhou leg. [1 spec., CS-340]; JZ, 26 VII 2002, Dandan Wang leg. [4 spec., CS-341–CS-343, CS-345]; JZ, 27 VII 2002, Dandan Wang leg. [1 spec., CS-344]; JZ, 25 VII 2002, Huixing Zhou leg. [2 spec., CS-346, CS-349]; JZ, 27 VII 2002, Huixing Zhou leg. [2 spec., CS-347, CS-348]; JZ, 24 VII 2002, Huixing Zhou leg. [1 spec., CS-350]; JZ, 26 VII 2002, Juan Zhang leg. [2 spec., CS-351, CS-352]; JZ, 28 VII 2002, Juan Zhang leg. [3 spec., CS-353–CS-355]; JZ, 27 VII 2002, Xiaoyan Wang leg. [2 spec., CS-356, CS-357]; JZ, 29 VII 2002, Xiaoyan Wang leg. [1 spec., CS-358]; JZ, 26 VII 2002, Taiyun Ji leg. [1 spec., CS-359]; JZ, 27 VII 2002, Feiyong Wang leg. [1 spec., CS-360]; JZ, 26 VII 2002, Xiaoqing Yu leg. [1 spec., CS-361]; JZ, 27 VII 2002, Yong Zhou leg. [1 spec., CS-362]; JZ, 28 VII 2002, Chaowu Liu leg. [1 spec., CS-363]; JZ, 22 VII 2002, Hurong Pei leg. [1 spec., CS-364]; JZ, 22 VII 2002, Jun Guo leg. [1 spec., CS-365]; JZ, 22 VII 2002, Xiaoqing Yu leg. [1 spec., CS-366]; JZ, 24 VII 2002, Xinhou Zhang leg. [1 spec., CS-367]; JZ, 27 VII 2002, Zhengyu Xiang leg. [2 spec., CS-368, CS-369]; JZ, 26 VII 2002, Ying Shi leg. [1 spec., CS-370]; JZ, 27 VII 2002, Ying Shi leg. [1 spec., CS-371]; JZ, 26 VII 2002, Huifang Li leg. [2 spec., CS-372, CS-373]; JZ, 28 VII 2002, Huifang Li leg. [2 spec., CS-374, CS-375]; JZ, 28 VII 2002, Tao Wan leg. [2 spec., CS-376, CS-377]; JZ, 25 VII 2002, Shuai Yan leg. [4 spec., CS-378–CS-381]; JZ, 28 VII 2002, Shuai Yan leg. [1 spec., CS-382]; JZ, 26 VII 2002, Ping Shen leg. [2 spec., CS-383, CS-384]; JZ, 25 VII 2002, Shaowei Wang leg. [1 spec., CS-385]; JZ, 26 VII 2002, Shaowei Wang leg. [2 spec., CS-386, CS-387]; JZ, 25 VII 2002, Pengbo Luo leg. [2 spec., CS-388, CS-389]; JZ, 26 VII 2002, Pengbo Luo leg. [2 spec., CS-390, CS-391]; JZ, 22 VII 2002, Yumin Yang leg. [1 spec., CS-392]; JZ, 24 VII 2002, Yumin Yang leg. [2 spec., CS-393, CS-394]; JZ, 25 VII 2002, Yumin Yang leg. [1 spec., CS-395]; JZ, 27 VII 2002, Yumin Yang leg. [1 spec., CS-396]; JZ, 28 VII 2002, Taiyun Ji leg. [1 spec., CS-397]; JZ, 26 VII 2002, Shuangxi Zhou leg. [1 spec., CS-398]; JZ, 21 VII 2002, Shuai Yan leg. [1 spec., CS-571]; JLC, 24 VII 1987, [1 spec., CS-593]; LPT, 2 VII 2012, 900 m a.s.l., Yangyang Xie leg. [1 spec., CS-586]; MYT, 12 VIII 2004, 1,200 m a.s.l., Jun Huang leg. [1 spec., CS-511]; MYT, 12 VIII 2004, 1,200 m a.s.l., Fengling Zou leg. [1 spec., CS-512]; MYT, 12 VIII 2004, 1,200 m a.s.l., Jingyang Peng leg. [1 spec., CS-513]; RHPT, 10 VII 2011, 600 m a.s.l., Wenpei Tian leg. [1 spec., CS-585]; SBT, 19 VII 2010, 900–1,300 m a.s.l., Pengfei Quan leg. [1 spec., CS-587]; SS, 13 VII 2008, Nian Liu leg. [1 spec., CS-576]; SS, 14 VII 2008, Jiaojiao Li leg. [1 spec., CS-577]; SS, 14 VII 2008, Feifei Song leg. [1 spec., CS-578]; SS, 17 VII 2008, Yi Bao leg. [1 spec., CS-579]; SYST, 12 VIII 2004, 1,000 m a.s.l., Panpan Wang leg. [1 spec., CS-510]; TSP, 15 VII 2006, 1,500 m a.s.l., Shaolan Liu leg. [1 spec., CS-573]; TSP, 14 VII 2006, 1,500 m a.s.l., Shaolan Liu leg. [1 spec., CS-580]; TSP, 14 VII 2006, 1,500 m a.s.l., Weiwei Xiang leg. [1 spec., CS-581]; TZS, 11 VII 2003, Shuang Gao leg. [1 spec., CS-595]; WJV: 13 VII 2009, Jianming Miao leg. [1 spec., CS-582]; WJV: 15 VII 2009, Jianming Miao leg. [1 spec., CS-583]; WSFF, 16 VII 2003, 1,700–2,000 m a.s.l., Fan Zhang leg. [1 spec., CS-588]; WSFF, 17 VII 2003, 1,700–2,000 m a.s.l., Liang Zhang leg. [1 spec., CS-589]; YPT, 13 VII 2009, Zhihui You leg. [1 spec., CS-584].

**Figures 26–34.**
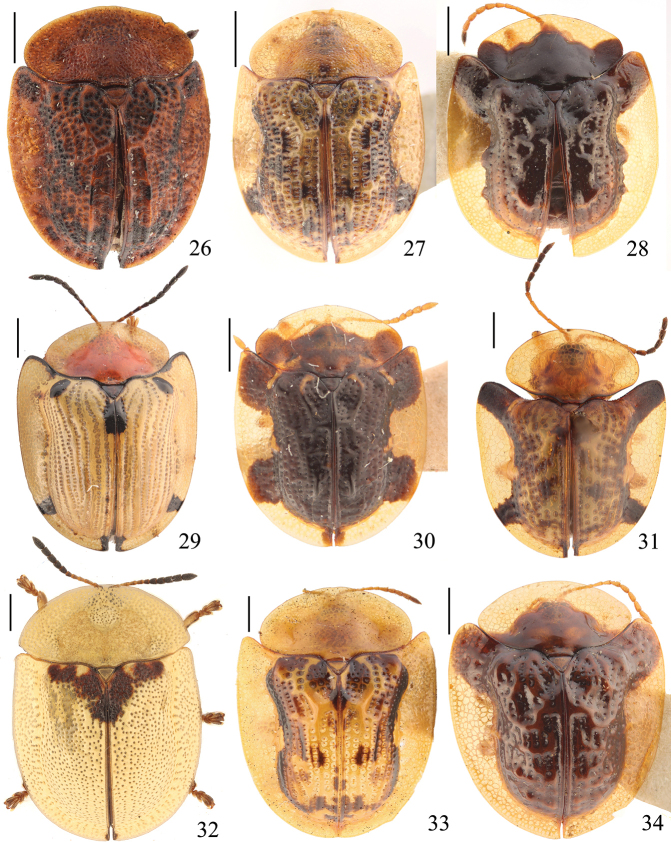
Specimens of Cassidinae deposited in the Insect Collection of the College of Agriculture, Yangtze University **26***Cassida pallidicollis* Boheman, 1856 **27***Cassida piperata* Hope, 1842 **28***Cassida postarcuata* (Chen & Zia, 1964) **29***Cassida purpuricollis* (Spaeth, 1914) **30***Cassida quinaria* (Chen & Zia, 1964) **31***Cassida rati* Maulik, 1923 **32***Cassida rubiginosa* Müller, 1776 **33***Cassida sauteri* (Spaeth, 1913) **34***Cassida sigillata* (Gorham, 1885). Scale bars: 1 mm.

There is no collection information for specimens CS-596 and CS-597.

**Distribution.** China (Beijing, Chongqing, Fujian, Guangdong, Guangxi, Hebei, Heilongjiang, Henan, Hubei, Jilin, Jiangsu, Jiangxi, Liaoning, Shaanxi, Shandong, Shanghai, Sichuan, Taiwan, Tianjin, Yunnan, Zhejiang); Japan; North Korea; Philippines; Russia (Far East); Vietnam.

#### ﻿*Cassida postarcuata* (Chen & Zia, 1964)

Fig. 28

**Material examined. Hubei**: WSFF, 16 VII 2003, 1700–2000 m a.s.l., Hua He leg. [1 spec., CS-097]; WSFF, 16 VII 2003, 1700–2000 m a.s.l., Suran Wu leg. [1 spec., CS-098].

**Distribution.** China (Fujian, Guizhou, Hubei*, Sichuan).

#### ﻿*Cassida purpuricollis* (Spaeth, 1914)

Fig. 29

**Material examined. Sichuan**: FPV, 26 VII 2024, 1,694.8 m a.s.l., 32.685468°N, 104.076105°E, Chaokun Yang leg. [3 spec., CS-640–CS-642]; FPV, 26 VII 2024, 1,717.2 m a.s.l., 32.686682°N, 104.076656°E, Chaokun Yang leg. [10 spec., CS-643–CS-652]; FPV, 22 VII 2024, 1,783.1 m a.s.l., 32.685896°N, 104.058208°E, Chaokun Yang leg. [2 spec., CS-638, CS-639].

**Distribution.** China (Guizhou, Hubei, Sichuan, Yunnan); Vietnam.

#### ﻿*Cassida quinaria* (Chen & Zia, 1964)

Fig. 30

**Material examined. Hubei**: HH, 15 VII 2002, Shaowei Wang leg. [1 spec., CS-115].

**Distribution.** China (Chongqing, Guangxi, Hubei*, Sichuan, Yunnan); Laos; Thailand; Vietnam.

#### ﻿*Cassida rati* Maulik, 1923

Fig. 31

**Material examined. Guizhou**: TLV, 25 VIII 2024, Chaokun Yang leg. [1 spec., CS-679]; **Hubei**: FXT, 12 VII 2017, 800 m a.s.l., 31.91028°N, 109.76444°E, Zhixiong Zhou leg. [1 spec., CS-601]; LWCV, 3 VII 2015, Ni Cai leg. [1 spec., CS-143]; XRX, 25 VI 2009, Guanglin Xie leg. [1 spec., CS-142].

**Distribution.** China (Fujian, Guangdong, Guangxi, Guizhou, Hubei*, Hunan, Jiangxi, Sichuan, Taiwan, Yunnan, Zhejiang); India; Indonesia; Laos; Myanmar; Vietnam.

#### ﻿*Cassida rubiginosa* Müller, 1776

Fig. 32

**Material examined. Hubei**: SBT, 17 VII 2003, 900–1,300 m a.s.l., Pengfei Quan leg. [1 spec., CS-135]; WSFF, 19 VII 2003, 1,700–2,000 m a.s.l., Wenkai Wang leg. [1 spec., CS-136]; WSFF, 20 VII 2003, 1,700–2,000 m a.s.l., Tiezheng Liu leg. [1 spec., CS-137]; WSFF, 18 VII 2003, 1,700–2,000 m a.s.l., He Zheng leg. [1 spec., CS-138]; WSFF, 22 VII 2003, 1,700–2,000 m a.s.l., Hua He leg. [1 spec., CS-139]; WSFF, 16 VII 2003, 1,700–2,000 m a.s.l., Hua He leg. [1 spec., CS-140]; WSFF, 20 VII 2003, 1,700–2,000 m a.s.l., Qinghua Zhou leg. [1 spec., CS-141]; **Sichuan**: FPT, 8 VIII 2024, Ping Wang leg. [1 spec., CS-677].

**Distribution.** China (Fujian, Hubei, Jiangsu, Qinghai, Shaanxi, Shanxi, Sichuan*, Taiwan, Xinjiang, Xizang, Zhejiang); Canada; Japan; North Korea; Russia.

#### ﻿*Cassida sauteri* (Spaeth, 1913)

Fig. 33

**Material examined. Chongqing**: MBV, 22 VII 2024, 1,413.3 m a.s.l., Liang Zhang, Jialing Chen, Wenbo Liang & Zixiang Wang leg. [1 spec., CS-609]; JFS, 23 VII 2024, 922.7 m a.s.l., Liang Zhang, Jialing Chen, Wenbo Liang & Zixiang Wang leg. [1 spec., CS-608]; **Guizhou**: KKS, 11 VIII 2024, Chaokun Yang leg. [8 spec., CS-311–CS-318]; KKS, 13 VIII 2024, 28.2345°N, 107.1588°E, Chaokun Yang leg. [3 spec., CS-604–CS-606]; KKS, 11 VIII 2024, Chaokun Yang leg. [7 spec., CS-670–CS-676]; **Hubei**: DLLNNR, 8 VII 2011, 1,200 m a.s.l., Xunsong Guan, [1 spec., CS-213]; HH, 18 VII 2002, Caihua Shi leg. [1 spec., CS-210]; JZ, 27 VII 2002, Xiaoyan Wang leg. [1 spec., CS-211]; JZ, 29 VII 2002, Xiaoyan Wang leg. [1 spec., CS-212]; QXT, 10 VII 2017, 860 m a.s.l., 32.08°N, 109.66389°E, Zhixiong Zhou leg. [1 spec., CS-214]; QXT, 10 VII 2017, 771 m a.s.l., 30.07611°N, 109.66278°E, Jiabo Wang leg. [1 spec., CS-215]; QXT, 13 VII 2017, 856 m a.s.l., 32.06861°N, 109.66139°E, Panlong Guo leg. [2 spec., CS-216, CS-217].

**Distribution.** China (Chongqing*, Fujian, Guangxi, Guizhou, Jiangxi, Sichuan, Taiwan, Yunnan, Zhejiang); Japan; Vietnam.

#### ﻿*Cassida sigillata* (Gorham, 1885)

Fig. 34

**Material examined. Guizhou**: XJ, 7 VI 2007, 630–650 m a.s.l., Guanglin Xie leg. [2 spec., CS-096], Note: the label includes 2 specimens. **Hubei**: HH, 27 IV 2004, 900–1,100 m a.s.l., Wenkai Wang leg. [2 spec., CS-083, CS-084]; HH, 28 IV 2004, 900–1,100 m a.s.l., Wenkai Wang leg. [2 spec., CS-085, CS-086]; HH, 30 IV 2004, 900–1,100 m a.s.l., Wenkai Wang leg. [1 spec., CS-087]; HH, 1 V 2004, 900–1,100 m a.s.l., Wenkai Wang leg. [1 spec., CS-088]; HH, 27 IV 2004, 900–1,100 m a.s.l., Guanglin Xie leg. [1 spec., CS-089]; HH, 29 IV 2004, 900–1,100 m a.s.l., Guanglin Xie leg. [1 spec., CS-090]; HH, 30 IV 2004, 900–1,100 m a.s.l., Guanglin Xie leg. [3 spec., CS-091–CS-093]; HH, 1 V 2004, 900–1,100 m a.s.l., Guanglin Xie leg. [1 spec., CS-094]; HH, 2 V 2004, 900–1,100 m a.s.l., Guanglin Xie leg. [1 spec., CS-095].

**Distribution.** China (Fujian, Guangdong, Hubei*, Jiangsu, Jiangxi, Taiwan, Zhejiang); Japan; North Korea.

#### ﻿*Cassida versicolor* (Boheman, 1855)

Fig. 35

**Material examined. Guangxi**: BDV, 4 VIII 2024, Guanglin Xie leg. [1 spec., CS-613]; **Hubei**: HH, 27 IV 2004, Wenkai Wang leg. [1 spec., CS-126]; MPT, 13 VII 2009, Xiaozhen Lu leg. [1 spec., CS-127]; MPT, 13 VII 2009, Beiyi Guo leg. [1 spec., CS-598]; MPT, 13–17 VII 2018, Li Mao leg. [1 spec., CS-128]; WJV, 13 VII 2009, Zhiqiang Ren leg. [1 spec., CS-129].

**Distribution.** China (Fujian, Guangdong, Guangxi, Guizhou, Hainan, Heilongjiang, Hubei, Hunan, Jiangxi, Sichuan, Taiwan, Yunnan, Zhejiang); Japan; Laos; Myanmar; North Korea; Russia (Far East); Vietnam.

#### ﻿*Cassida vespertina* Boheman, 1862

Fig. 36

**Material examined. Chongqing**: MBV, 21 VII 2024, Liang Zhang, Jialing Chen, Wenbo Liang & Zixiang Wang leg. [1 spec., CS-653]; **Guangxi**: BNV, 12 VIII 2024, Guanglin Xie leg. [1 spec., CS-614]; **Hubei**: BJMV, 22 VII 2003, 900–1,300 m a.s.l., Hua Huang, [1 spec., CS-099]; BJMV, 18 VII 2003, 900–1,300 m a.s.l., Yang Liu leg. [1 spec., CS-105]; BYT, 12 VII 2017, 491 m a.s.l., 32.11404°N, 109.94912°E, Zhenzhou Xia leg. [1 spec., CS-114]; HH, 2 V 2004, Wenkai Wang leg. [1 spec., CS-108]; HHT, 16 VII 2009, Guanwei Deng leg. [1 spec., CS-102]; JZ, 22 VII 2002, Jun Guo leg. [1 spec., CS-103]; MPT, 13 VII 2009, Wenjing An leg. [1 spec., CS-106]; MPT, 14 VII 2009, Zhou Deng leg. [1 spec., CS-107]; NJHT, 13 VII 2008, Xianhui Zhang leg. [1 spec., CS-100]; NJHT, 13 VII 2008, Tao Yan leg. [1 spec., CS-101]; QGP, 11 VII 2014, Yin Luo leg. [1 spec., CS-104]; QXT, 10 VII 2017, 812 m a.s.l., 32.07278°N, 109.66028°E, Jiabo Wang leg. [1 spec., CS-113]; SYST, 12 VII 2004, Lan Zhang leg. [1 spec., CS-109]; TZS, 13 VII 2005, Yu’e Chen leg. [1 spec., CS-110]; XBT, 5 VII 2017, 1,208 m a.s.l., 31.5641°N, 109.86863°E, Zhenzhou Xia leg. [1 spec., CS-111]; XBT, 5 VII 2017, 1,156 m a.s.l., 31.56278°N, 109.89806°E, Jiabo Wang leg. [1 spec., CS-112].

**Figures 35–40.**
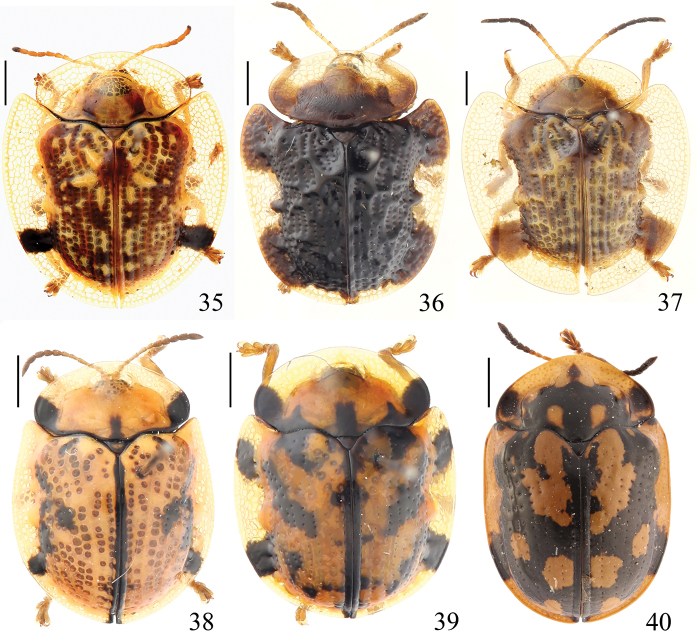
Specimens of Cassidinae deposited in the Insect Collection of the College of Agriculture, Yangtze University **35***Cassida versicolor* (Boheman, 1855) **36***Cassida vespertina* Boheman, 1862 **37***Thlaspida biramosa* (Boheman, 1855) **38**Glyphocassis (Hebdomecosta) lepida (Spaeth, 1914) **39**Glyphocassis (Hebdomecosta) spilota (Gorham, 1885) **40**Glyphocassis (s. str.) trilineata (Hope, 1831). Scale bars: 1 mm.

**Distribution.** China (Beijing, Chongqing*, Fujian, Gansu, Guangdong, Guangxi, Guizhou, Hebei, Heilongjiang, Hubei, Hunan, Inner Mongolia, Jiangsu, Jiangxi, Shaanxi, Sichuan, Taiwan, Zhejiang); Japan; Mongolia; North Korea; Russia (Far East).

### ﻿Genus *Thlaspida* Weise, 1899

#### ﻿*Thlaspida biramosa* (Boheman, 1855)

Fig. 37

**Material examined. Guangdong**: NKS, 16 V 2007, Liangkui Tan leg. [2 spec., CS-132, CS-133]; **Guangxi**: CFV, 2 VIII 2024, 828 m a.s.l., 24.9402°N, 107.7574°E, Guanglin Xie leg. [1 spec., CS-615]; XCV, 10 VIII 2024, 960 m a.s.l., 24.84°N, 106.31°E, Guanglin Xie leg. [4 spec., CS-616–CS-619]; YG, 8 VIII 2024, 389 m a.s.l., 24.81°N, 106.19°E, Guanglin Xie leg. [2 spec., CS-620, CS-621]; YG, 17 IV 2024, 470 m a.s.l., 24.7979°N, 106.2125°E, Guanglin Xie leg. [2 spec., CS-622, CS-623]; **Guizhou**: NPT, 18 VIII 2024, Chaokun Yang leg. [3 spec., CS-308–CS-310]; **Hubei**: LWCV, 3 VII 2015, Ru Yan leg. [1 spec., CS-131]; SDS, 11 VII 2005, Bo Shi leg. [1 spec., CS-134]; WJV, 13 VII 2009, Cui Ye leg. [1 spec., CS-130].

**Distribution.** China (Anhui, Fujian, Guangdong, Guangxi, Guizhou, Hainan, Hubei, Hunan, Jiangsu, Jiangxi, Sichuan, Taiwan, Yunnan, Zhejiang); India; Japan; Laos; Malaysia; Myanmar; North Korea; Thailand; Vietnam.

### ﻿Genus *Glyphocassis* Spaeth, 1914

#### ﻿Glyphocassis (Hebdomecosta) lepida (Spaeth, 1914)

Fig. 38

**Material examined. Hubei**: HH, 19 VII 2002, Hongmei Zhang leg. [1 spec., CS-021]; HH, 15 VII 2002, Taiyun Ji leg. [1 spec., CS-032]; LWCV, 2 VII 2015, Qi Zhang leg. [1 spec., CS-017]; LWCV, 4 VII 2015, Xiaoling Li leg. [1 spec., CS-022]; LWCV, 6 VII 2015, Yu Chen leg. [1 spec., CS-023]; LWCV, 2 VII 2015, Jianbin Lv leg. [1 spec., CS-024]; LWCV, 5 VII 2015, Ni Cai leg. [1 spec., CS-029; MYT, 12 VII 2004, 900 m a.s.l., Zhen Wang leg. [1 spec., CS-025]; MYT, 12 VII 2004, 900 m a.s.l., Ying Zhang leg. [1 spec., CS-026]; QGPV, 2–7 VII 2023, Jing Li & Weijia Yang leg. [1 spec., CS-016]; QGPV, 11 VII 2014, Chunjiao Jin leg. [1 spec., CS-030]; QGPV, 13–17 VII 2018, Wenchao Sun leg. [1 spec., CS-033]; QGPV, 11 VII 2014, Zhiqin Chen leg. [1 spec., CS-036]; SBT, 18 VII 2003, 900–1,300 m a.s.l., Yinghua He, et. al leg. [1 spec., CS-031]; SQPV, 1–6 VII 2023, Liqing Yuan & Qian Shui leg. [1 spec., CS-034]; SYST, 12 VII 2004, Lan Zhang leg. [1 spec., CS-019]; TZS, 12 VII 2015, Hui Xiong leg. [1 spec., CS-018]; XBT, 6 VII 2017, 1,130 m a.s.l., 31.57611°N, 109.8525°E, Zhixiong Zhou leg. [1 spec., CS-015]; XBT, 9 VII 2017, 1,102 m a.s.l., 31.59206°N, 109.82312°E, Zhenzhou Xia leg. [1 spec., CS-035]; ZGW, 13 VII 2005, Jiaqing Deng leg. [1 spec., CS-020]; ZGW, 11 VII 2005, Shuibing Lao leg. [1 spec., CS-027]; ZGW, 15 VII 2005, Youting Li leg. [1 spec., CS-028]; **Sichuan**: FPV, 26 VII 2024, 1,717.2 m a.s.l., 32.686682°N, 104.076656°E, Chaokun Yang leg. [1 spec., CS-628]; FPV, 26 VII 2024, 1,694.8 m a.s.l., 32.685468°N, 104.076105°E, Chaokun Yang leg. [1 spec., CS-629]; SG, 23 VII 2024, 1,783.1 m a.s.l., 32.685896°N, 104.058208°E, Chaokun Yang leg. [2 spec., CS-626, CS-627].

**Distribution.** China (Hubei, Jiangxi, Sichuan).

#### ﻿Glyphocassis (Hebdomecosta) spilota (Gorham, 1885)

Fig. 39

**Material examined. Chongqing**: MBV, 22 VII 2024, 1,413.3 m a.s.l., Liang Zhang, Jialing Chen, Wenbo Liang & Zixiang Wang leg. [3 spec., CS-305–CS-307]; **Guizhou**: CMC, 19 VIII 2024, 668 m a.s.l., Chaokun Yang leg. [2 spec., CS-303, CS-304]; KKS, 13 VIII 2024, 28.2345°N, 107.1588°E, Chaokun Yang leg. [1 spec., CS-607]; **Hubei**: BJMV, 19 VII 2003, 900–1,300 m a.s.l., Songlin Li leg. [1 spec., CS-064]; BYT, 12 VII 2017, 530 m a.s.l., 32.14239°N, 109.92029°E, Panlong Guo leg. [1 spec., CS-073]; BYT, 13 VII 2017, 856 m a.s.l., 32.06861°N, 109.66139°E, Panlong Guo leg. [1 spec., CS-074]; CLPT, 13 VII 2008, Liang Yu leg. [1 spec., CS-039]; DLLFF, 26 VII 2010, Peng Cheng leg. [1 spec., CS-079]; HH, 16 VII 2002, Dingzhi Xiong leg. [1 spec., CS-044]; HH, 15 VII 2002, Shouhe Yu leg. [1 spec., CS-045]; HH, 19 VII 2002, Wenkai Wang leg. [1 spec., CS-049]; HH, 16 VII 2002, Taiyun Ji leg. [1 spec., CS-050]; HH, 15 VII 2002, Pei Zhong leg. [1 spec., CS-051]; HH, 16 VII 2002, Tianpeng Zhang leg. [1 spec., CS-052]; HH, 14 VII 2002, Linjun Hong leg. [1 spec., CS-053]; HH, 19 VII 2002, Jianbo Yan leg. [1 spec., CS-054]; HH, 18 VII 2002, Xiaowei Zhang leg. [1 spec., CS-058]; HH, 1 V 2004, 900–1,100 m a.s.l., Guanglin Xie leg. [1 spec., CS-059]; HH, 15 VII 2002, Shouhe Yu leg. [1 spec., CS-067]; HLT, 14 VIII 2004, 1,000 m a.s.l., Yiping Zou leg. [1 spec., CS-041]; HWT, 15 VII 2017, 417 m a.s.l., 32.10889°N, 109.81444°E, Jiabo Wang leg. [1 spec., CS-075]; JJYT, 17 VII 2017, 650 m a.s.l., 32.32838°N, 109.51211°E, Zhenzhou Xia leg. [1 spec., CS-076]; JZ, 28 VII 2002, Shuangxi Zhou leg. [1 spec., CS-043]; JZ, 23 VII 2002, Xiaopan Yu leg. [1 spec., CS-055]; JZ, 22 VII 2002, Xiaoqing Yu leg. [1 spec., CS-066]; LBT, 20 IV 2017, 513 m a.s.l., 32.3675°N, 109.62222°E, Chuanren Li leg. [1 spec., CS-046]; LWCV, VII 2015, Ru Yan leg. [1 spec., CS-048]; LWCV, 3 VII 2015, Ni Cai leg. [1 spec., CS-060]; LWCV, 1 VII 2015, Yuxin Deng leg. [1 spec., CS-081]; QGPV, 7 VII 2017, Dan Yang leg. [1 spec., CS-063]; QXT, 10 VII 2017, 968 m a.s.l., 31.56917°N, 109.86833°E, Panlong Guo leg. [1 spec., CS-070]; SBT, 21 VII 2003, 900–1,300 m a.s.l., Desheng Liu leg. [1 spec., CS-037]; SBT, 21 VII 2003, 900–1,300 m a.s.l., Jin Cheng leg. [1 spec., CS-047]; SBT, 18 VII 2003, 900–1,300 m a.s.l., Xiaowen Pei leg. [1 spec., CS-065]; SQPV, 1–6 VII 2023, Houze Hu leg. [1 spec., CS-078]; SYST, 12 VII 2004, Panpan Wang leg. [1 spec., CS-040]; TBT, 11 VII 2017, 518 m a.s.l., 32.045°N, 109.86528°E, Xin Liu leg. [1 spec., CS-071]; TBT, 11 VII 2017, 458 m a.s.l., 31.91167°N, 109.80389°E, Zhixiong Zhou leg. [1 spec., CS-072]; TYT, 10 VII 2017, 853 m a.s.l., 31.88071°N, 109.96117°E, Zhenzhou Xia leg. [1 spec., CS-068]; TYT, 10 VII 2017, 764 m a.s.l., 31.75245°N, 109.86339°E, Zhenzhou Xia leg. [1 spec., CS-069]; TZS, 11 VII 2005, Yanbing Guo leg. [1 spec., CS-056]; TZS, 11 VII 2005, Xia Wang leg. [1 spec., CS-080]; TZS, 11 VII 2005, Yan Peng leg. [1 spec., CS-082]; WJV, 14 VII 2009, Kun Wang leg. [1 spec., CS-057]; WJV, 12 VII 2009, Chao Liang leg. [1 spec., CS-061]; WJV, 13 VII 2009, Wei Zhou leg. [1 spec., CS-062]; XPV, 20 VI 2008, 1,070 m a.s.l., Guanglin Xie leg. [1 spec., CS-038]; XZT, 19 VII 2017, 363 m a.s.l., 32.17319°N, 109.98983°E, Xin Liu leg. [1 spec., CS-077]; ZGW, 1 spec., 13 VII 2005, Chengzhi Wu leg. [1 spec., CS-042]; **Sichuan**: FPV, 26 VII 2024, 1,694.8 m a.s.l., 32.685468°N, 104.076105°E, Chaokun Yang leg. [1 spec., CS-625]; FPV, 23 VII 2024, 1,783.1 m a.s.l., 32.685896°N, 104.058208°E, Chaokun Yang leg. [1 spec., CS-626]; FPV, 22 VII 2024, 1,783.1 m a.s.l., 32.685896°N, 104.058208°E, Chaokun Yang leg. [1 spec., CS-627].

**Distribution.** China (Beijing, Chongqing*, Fujian, Gansu, Guizhou, Hebei, Heilongjiang, Hubei*, Hunan, Jilin, Jiangsu, Liaoning, Shandong, Shanghai, Sichuan, Zhejiang); Japan; North Korea; Russia (Far East); Vietnam.

#### ﻿Glyphocassis (s. str.) trilineata (Hope, 1831)

Fig. 40

**Material examined. Hubei**: HH, 20 VII 2002, Ping Shen leg. [1 spec., CS-004]; HH, 17 VII 2002, Xinhou Zhang leg. [1 spec., CS-007]; HH, 19 VII 2002, Xinhou Zhang leg. [1 spec., CS-008]; JZ, 28 VII 2002, Xiaoyan Wang leg. [1 spec., CS-006]; JZ, 26 VII 2002, Jingrong Zheng leg. [1 spec., CS-009]; NJHT, 17 VII 2008, Xiang Zhao leg. [1 spec. CS-012]; NMPV, 15 VII 2005, Qifeng Fan leg. [1 spec. CS-013]; RHPT, 9 VII 2011, 600 m a.s.l., Wenpei Tian leg. [1 spec., CS-003]; RHPT, VII–XI 2013, Linxiang Chen leg. [1 spec., CS-005]; TZS, 12 VII 2005, Fuxiang Hu leg. [1 spec., CS-002]; TZS, 15 VII 2005, Yuyuan Chen leg. [1 spec., CS-011]; ZGW, 12 VII 2005, Lei Li leg. [1 spec., CS-001]; ZGW, 13 VII 2005, Zhengzhuan Xu leg. [1 spec., CS-010]; ZGW, 11 VII 2005, Zhengzhuan Xu leg. [1 spec., CS-014].

**Distribution.** China (Chongqing, Guangxi, Guizhou, Hubei, Sichuan, Yunnan); India; Laos; Nepal; Pakistan; Thailand; Vietnam.

### ﻿Tribe Gonophorini Chapuis, 1875

**Genus *Agonita* Strand, 1942**

#### ﻿*Agonita maculigera* (Gestro, 1888)

Fig. 41

**Material examined. Hainan**: DLS, 29 V 2010, Wei Li leg. [1 spec., G-001].

**Distribution.** China (Fujian, Guangdong, Hainan, Yunnan); Cambodia; India; Laos; Myanmar; Nepal; Thailand; Vietnam.

### ﻿Tribe Hispini Gyllenhal, 1813

**Genus *Dactylispa* Weise, 1897**

#### ﻿*Dactylispa angulosa* (Solsky, 1871)

Fig. 42

**Material examined. Hubei**: HBAU, 27 VIII 2001, [1 spec., H-015]; HBAU, 27 VIII 2001, [1 spec., H-045]; HH, 17 VII 2002, Shouhe Yu leg. [1 spec., H-002]; HH, 19 VII 2002, Taiyun Ji leg. [1 spec., H-003]; HH, 20 VII 2002, Chaoyun Liu leg. [1 spec., H-004]; HH, 20 VII 2002, Linling Li leg. [2 spec., H-006, H-011]; HH, 20 VII 2002, Shouhe Yu leg. [[spec., H-007]; HH, 16 VII 2002, Pei Zhong leg. [1 spec., H-008]; HH, 19 VII 2002, Ying Shi leg. [1 spec., H-009]; HH, 16 VII 2002, Hongmei Zhang leg. [1 spec., H-012]; HH, 3 V 2004, Wenkai Wang leg. [1 spec., H-019]; HH, 16 VII 2002, Linjun Hong leg. [2 spec., H-023, H-053]; HH, 18 VII 2002, Taiyun Ji leg. [1 spec., H-024]; HH, 21 VII 2002, Pei Zhong leg. [1 spec., H-027]; HH, 20 VII 2002, Pei Zhong leg. [1 spec., H-028]; HH, 17 VII 2002, Pei Zhong leg. [1 spec., H-029]; HH, 18 VII 2002, Taiyun Ji leg. [1 spec., H-030]; HH, 15 VII 2002, Min Liu leg. [2 spec., H-031, H-051]; HH, 15 VII 2002, Pei Zhong leg. [1 spec., H-032]; HH, 16 VII 2002, Hongmei Zhang leg. [1 spec., H-033]; HH, 16 VII 2002, Shouhe Yu leg. [3 spec., H-034, H-056, H-065]; HH, 16 VII 2002, Li Wang leg. [1 spec., H-035]; HH, 18 VII 2002, Xuguang Tang leg. [1 spec., H-036]; HH, 16 VII 2002, Xiaoyan Wang leg. [1 spec., H-037]; HH, 20 VII 2002, Linling Li leg. [1 spec., H-039]; HH, 19 VII 2002, Ying Shi leg. [2 spec., H-042, H-067]; HH, 19 VII 2002, Kaiqing Huang leg. [1 spec., H-043]; HH, 14 VII 2002, Shouhe Yu leg. [1 spec., H-046]; HH, 19 VII 2002, Linling Li leg. [2 spec., H-048, H-057]; HH, 21 VII 2002, Zhuangli Xiang leg. [1 spec., H-049]; HH, 15 VII 2002, Zhuangli Xiang leg. [1 spec., H-050]; HH, 27 VII 2002, Taiyun Ji leg. [1 spec., H-052]; HH, 18 VII 2002, Linjun Hong leg. [1 spec., H-054]; HH, 19 VII 2002, Shouhe Yu leg. [1 spec., H-055]; HH, 15 VII 2002, Pei Zhou leg. [1 spec., H-058]; HH, 21 VII 2002, Hancheng Wang leg. [1 spec., H-059]; HH, 17 VII 2002, Shouhe Yu leg. [1 spec., H-060]; HH, 20 VII 2002, Linling Li leg. [1 spec., H-062]; HH, 18 VII 2002, Hongmei Zhang leg. [1 spec., H-064]; HH, 14 VII 2002, Linjun Hong leg. [1 spec., H-066]; JZ, 24 VII 2002, Xiangqian Hu leg. [2 spec., H-005, H-010]; JZ, 24 VII 2002, Xiaoqing Yu leg. [1 spec., H-021]; JZ, 5 VII 2002, Qiyun Xu leg. [1 spec., H-022]; JZ, 24 VII 2002, Dingzhi Xiong leg. [1 spec., H-025]; JZ, 29 VII 2002, Xinhou Xiong leg. [1 spec., H-026]; JZ, 24 VII 2002, Ma Li leg. [1 spec., H-047]; JZ, 29 VII 2002, Feiyong Wang leg. [1 spec., H-061; JZ, 24 VII 2002, Xiangqian Hu leg. [1 spec., H-063]; NJHT, 15 VII 2008, Lijuan Cong leg. [1 spec., H-001]; QXT, 1 VII 2017, 952 m a.s.l., 32.03167°N, 109.68667°E, Zhixiong Zhou leg. [1 spec., H-072]; QXT, 11 VII 2017, 1,190 m a.s.l., 32.03167°N, 109.68667°E, Zhenzhou Xia leg. [1 spec., H-074]; SBT, 19 VII 2003, 900–1,300 m a.s.l., Wei Wang leg. [1 spec., H-018]; SBT, 17 VII 2003, 900–1,300 m a.s.l., Linling Li leg. [1 spec., H-044]; SDS, 11 VII 2005, Manli Wei leg. [1 spec., H-013]; SDS, 12 VII 2005, Zewen Wang leg. [1 spec., H-014]; SDS, 13 VII 2005, Bo Shi leg. [1 spec., H-017]; SZPT, 19 VII 2011, 400 m a.s.l., Wanwen Zheng leg. [1 spec., H-069]; TBT, 11 VII 2017, 458 m a.s.l., 31.91167°N, 109.80389°E, Zhixiong Zhou leg. [1 spec., H-073]; WJV, 16 VII 2009, Yujie Deng leg. [1 spec., H-068]; WJV, 19 V 1994, [1 spec., H-070]; XBPT, 13 VIII 2004, Hongyan Lei leg. [1 spec., H-016]; XBPT, 12 VIII 2004, Hongyan Lei leg. [1 spec., H-020]; XBPT, 12 VII 2004, 1,000 m a.s.l., Yanfang Xiao leg. [1 spec., H-040]; ZGW, 13 VII 2003, Youting Li leg. [1 spec., H-038]; ZGW, 15 VII 2003, Youting Li leg. [1 spec., H-041]; **Sichuan**: FPV, 26 VII 2024, 1,732.5 m a.s.l., Chaokun Yang leg. [1 spec., H-106].

There is no collection information for specimens (H-071).

**Distribution.** China (Anhui, Beijing, Fujian, Gansu, Guangdong, Guangxi, Guizhou, Hebei, Henan, Heilongjiang, Hubei, Hunan, Jilin, Jiangsu, Jiangxi, Liaoning, Shaanxi, Shanxi, Shandong, Shanghai, Sichuan, Taiwan, Tianjin, Yunnan, Zhejiang); Japan; North Korea; Russia (Siberia).

#### ﻿*Dactylispa chinensis* Weise, 1905

Fig. 43

**Material examined. Chongqing**: JFS, 26 VII 2024, 1,300 m a.s.l., Liang Zhang, Jialing Chen, Wenbo Liang & Zixiang Wang leg. [1 spec., H-097]; **Guizhou**: TLV, 24 VIII 2024, Chaokun Yang leg. [1 spec., H-111].

**Distribution.** China (Chongqing*, Fujian, Guangdong, Guangxi, Guizhou, Hainan, Hubei, Hunan, Jiangxi, Sichuan, Taiwan, Yunnan).

#### ﻿*Dactylispa crassicuspis* Gestro, 1906

Fig. 44

**Material examined. Hubei**: MYT, 12 VII 2003, 1200 m a.s.l., Shuibin Lao leg. [1 spec., H-078]; SBT, 17 VII 2003, 900–1300 m a.s.l., Junnan Wang leg. [1 spec., H-079].

**Distribution.** China (Fujian, Guangdong, Guizhou, Hubei, Hunan, Jiangxi, Shaanxi, Sichuan, Yunnan).

**Figures 41–49.**
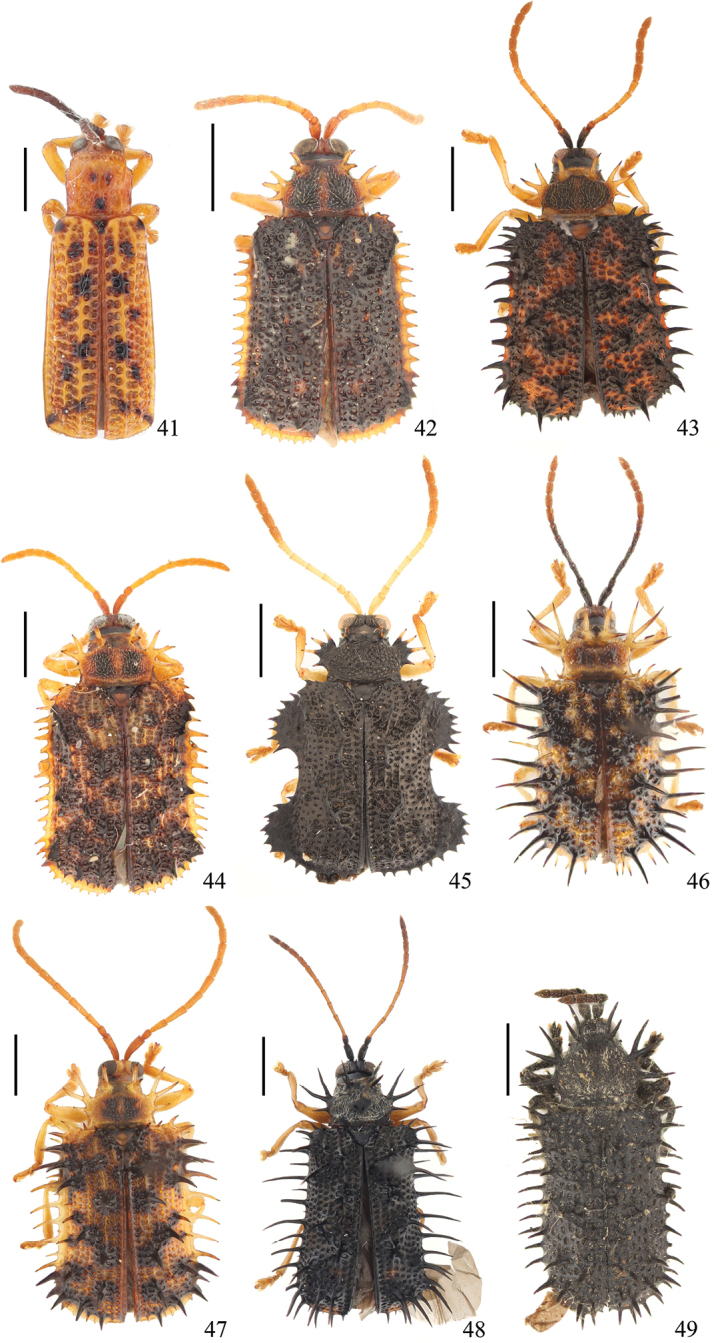
Specimens of Cassidinae deposited in the Insect Collection of the College of Agriculture, Yangtze University **41***Agonita maculigera* (Gestro, 1888) **42***Dactylispa angulosa* (Solsky, 1871) **43***Dactylispa chinensis* Weise, 1905 **44***Dactylispa crassicuspis* Gestro, 1906 **45***Dactylispa excisa* (Kraatz, 1879) **46***Dactylispa feae* Gestro, 1888 **47***Dactylispa higoniae* Lewis, 1896 **48***Dactylispa sauteri* Uhmann, 1927 **49***Hispellinus callicanthus* (Bates, 1866). Scale bars: 1 mm.

#### ﻿*Dactylispa excisa* (Kraatz, 1879)

Fig. 45

**Material examined. Chongqing**: JFS, 23 VII 2024, 922.7 m a.s.l., Liang Zhang, Jialing Chen, Wenbo Liang & Zixiang Wang leg. [1 spec., H-096]; **Hubei**: BYT, 12 VII 2017, 530 m a.s.l., 32.14239°N, 109.92029°E, Xihuan Zhou leg. [1 spec., H-077]; SYST, 12 VIII 2004, 1, 000 m a.s.l., Yongjiao Wang leg. [1 spec., H-076]; **Yunnan**: DL, 16 VIII 2008, Guanglin Xie leg. [1 spec., H-075].

**Distribution.** China (Anhui, Chongqing, Fujian, Guizhou, Guangdong, Guangxi, Heilongjiang, Hubei, Hunan, Jilin, Jiangxi, Shaanxi, Shandong, Sichuan, Taiwan, Yunnan, Zhejiang); Japan; North Korea; Russia; South Korea.

#### ﻿*Dactylispa feae* Gestro, 1888

Fig. 46

**Material examined. Guangxi**: YG, 8 VIII 2024, 389 m a.s.l., 24.81°N, 106.19°E, Guanglin Xie leg. [1 spec., H-101].

**Distribution.** China (Fujian, Guangdong, Guangxi, Hainan, Hunan, Jiangxi, Sichuan, Taiwan, Yunnan); Cambodia; India; Indonesia; Laos; Malaysia; Myanmar; Sri Lanka; Thailand; Vietnam.

#### ﻿*Dactylispa higoniae* Lewis, 1896

Fig. 47

**Material examined. Guizhou**: CMC, 19 VIII 2024, 685 m a.s.l., Chaokun Yang leg. [2 spec., H-107, H-108]; NPT, 18 VIII 2024, Chaokun Yang leg. [2 spec., H-112, H-113]; YK, 12 VIII 2024, Chaokun Yang leg. [2 spec., H-109, H-110].

**Distribution.** China (Fujian, Guangdong, Guangxi, Guizhou, Hainan, Hunan, Jiangxi, Sichuan, Taiwan, Xizang, Yunnan); Bhutan; India; Japan; Laos; Myanmar; Nepal; Thailand; Vietnam.

#### ﻿*Dactylispa sauteri* Uhmann, 1927

Fig. 48

**Material examined. Guizhou**: KKS, 11 VIII 2024, Chaokun Yang leg. [1 spec., H-095]; NPT, 18 VIII 2024, Chaokun Yang leg. [1 spec., H-114]; **Hubei**: DLLFF, 22 VII 2010, Kui Hu leg. [1 spec., H-083]; HH, 16 VII 2002, Xiaoyan Wang leg. [1 spec., H-080]; HH, 20 VII 2002, Ping Shen leg. [1 spec., H-081]; HH, 29 IV 2004, 900–1,100 m a.s.l., Wenkai Wang leg. [1 spec., H-082]; JZ, 24 VII 2002, Jun Guo leg. [1 spec., H-085]; TYT, 12 VII 2017, 769 m a.s.l., 31.75245°N, 109.86339°E, Zhenzhou Xia leg. [1 spec., H-084]; **Sichuan**: FPV, 26 VII 2024, 1,695.3 m a.s.l., 32.686504°N, 104.075323°E, Chaokun Yang leg. [3 spec., H-102–H-104]; FPV, 26 VII 2024, Chaokun Yang leg. [1 spec., H-105].

**Distribution.** China (Fujian, Guangdong, Guangxi, Guizhou, Hubei, Hunan, Jiangxi, Sichuan, Taiwan, Yunnan, Zhejiang).

### ﻿Genus *Hispellinus* Weise, 1897

#### ﻿*Hispellinus callicanthus* (Bates, 1866)

Fig. 49

**Material examined. Hubei**: HZS, 11 VII 2007, Jingkai Li leg. [1 spec., H-094].

**Distribution.** China (Anhui, Fujian, Guangdong, Guangxi, Guizhou, Hainan, Hubei, Hunan, Jiangsu, Jiangxi, Taiwan, Yunnan); Cambodia; India; Indonesia; Laos; Malaysia; Myanmar; Philippines; Sri Lanka; Thailand; Vietnam.

#### ﻿*Hispellinus chinensis* Gressitt, 1950

Fig. 50

**Material examined. Hubei**: HZS, 10 VII 2007, Jiao Xiang leg. [1 spec., H-092]; YJG, 16 VII 2006, Zhen Deng leg. [1 spec., H-093].

**Distribution.** China (Chongqing, Hubei*, Hunan, Jiangxi, Shandong, Sichuan); South Korea.

#### ﻿*Hispellinus moerens* (Baly, 1874)

Fig. 51

**Material examined. Hubei**: HH, 20 VII 2002, Ying Shi leg. [1 spec., H-087]; HH, 21 VII 2002, Linjun Hong leg. [1 spec., H-088]; HH, 16 VII 2002, Hongshan Cheng leg. [1 spec., H-090]; HH, 17 VII 2002, Yong Zhou leg. [1 spec., H-091]; JZ, 22 VII 2002, Hurong Pei leg. [1 spec., H-089].

**Distribution.** China (Hebei, Heilongjiang, Hubei*, Jiangsu, Jiangxi, Liaoning, Shandong, Shanghai, Taiwan, Tianjin, Zhejiang); Japan; North Korea; Russia (Siberia).

### ﻿Genus *Monohispa* Weise, 1897

#### ﻿*Monohispa tuberculata* Gressitt, 1950

Fig. 52

**Material examined. Guangdong**: NKS, 16 V 2007, Liangkui Tan leg. [1 spec., H-086].

**Distribution.** China (Fujian, Guangdong, Guangxi, Yunnan).

**Figures 50–53.**
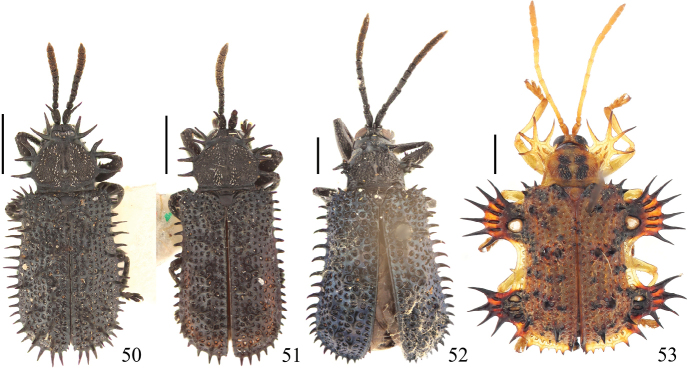
Specimens of Cassidinae deposited in the Insect Collection of the College of Agriculture, Yangtze University **50***Hispellinus chinensis* Gressitt, 1950 **51***Hispellinus moerens* (Baly, 1874) **52***Monohispa tuberculata* Gressitt, 1950 **53***Platypria aliena* Chen & Sun, 1962. Scale bars: 1 mm.

### ﻿Genus *Platypria* Guérin-Méneville, 1840

#### ﻿*Platypria aliena* Chen & Sun, 1962

Fig. 53

**Material examined. Guangxi**: YG, 8 VIII 2024, 389 m a.s.l., 24.81°N, 106.19°E, Guanglin Xie leg. [3 spec., H-098–H-100].

**Distribution.** China (Guangxi*, Yunnan).

## ﻿Discussion

The preliminary catalog presented here documents, as of October 2024, a total of 1,207 specimens belonging to 53 species, 13 genera, and six tribes of Cassidinae housed in YZU. Among the 53 species, the tribe Cassidini is the most diverse, with 24 species, accounting for 45.3% of the total species, followed by Hispini (12 species, 22.6%). In addition, 14 species were recorded for the first time in Hubei Province, five species in Chongqing, and one species each in Zhejiang, Guangxi, and Sichuan Provinces. The new species records provide important insights into the distribution and biodiversity of the Cassidinae in China. Some widely distributed species such as *Dactylispa chinensis* Weise, 1905, *Cassida rubiginosa* Müller, 1776, and Glyphocassis (Hebdomecosta) spilota (Gorham, 1885) also have new records, indicating that many potential habitats in China have not been fully studied (Liu et al. 2019; Yang et al. 2023; Yang et al. 2024). Considering that these newly recorded species may also exist in other similar environments, further investigations in the future will help fill the gaps in the distribution of species in various provinces and regions in China.

To our knowledge, no previous studies in China have systematically cataloged a single collection of Cassidinae specimens while also providing photographs of all documented species. Furthermore, since the publication of Fauna Sinica (Insecta: Coleoptera: Hispidae) (Chen et al. 1986), specialized revisions or regional catalogs dedicated to Cassidinae in China have remained relatively scarce (Qi 2009; Lee et al. 2011, 2012; Liu et al. 2019; Yang et al. 2023, 2024). Therefore, this study may represent the first comprehensive effort of its kind, providing a valuable reference for future taxonomic, biogeographic, and ecological research.

Many universities and research institutions across China may house additional Cassidinae specimens in their collections. Where possible, we will strengthen collaboration with these institutions to compile more comprehensive data and photograph additional species, contributing to the foundational research on Cassidinae in China.

## ﻿Additional information

### Author ORCIDs

Chaokun Yang https://orcid.org/0009-0009-9868-487X

Liang Zhang https://orcid.org/0009-0004-1718-1466

Ping Wang https://orcid.org/0000-0002-8852-601X

Guanglin Xie https://orcid.org/0000-0003-3273-8985

Wenkai Wang https://orcid.org/0000-0002-4482-076X

### Data availability

All of the data that support the findings of this study are available in the main text.
